# Arabidopsis CP12 mutants have reduced levels of phosphoribulokinase and impaired function of the Calvin–Benson cycle

**DOI:** 10.1093/jxb/erx084

**Published:** 2017-04-20

**Authors:** Patricia Elena López-Calcagno, Amani Omar Abuzaid, Tracy Lawson, Christine Anne Raines

**Affiliations:** 1 Department of Biological Sciences, University of Essex, Wivenhoe Park, Colchester CO4 3SQ, UK

**Keywords:** Chloroplast metabolism, CP12, phosphoribulokinase, photosynthesis, plant development, protein family, redox, T-DNA mutants.

## Abstract

CP12 is a small, redox-sensitive protein, the most detailed understanding of which is the thioredoxin-mediated regulation of the Calvin–Benson cycle, where it facilitates the formation of a complex between glyceraldehyde-3-phosphate dehydrogenase (GAPDH) and phosphoribulokinase (PRK) in response to changes in light intensity. In most organisms, CP12 proteins are encoded by small multigene families, where the importance of each individual CP12 gene *in vivo* has not yet been reported. We used *Arabidopsis thaliana* T-DNA mutants and RNAi transgenic lines with reduced levels of CP12 transcript to determine the relative importance of each of the CP12 genes. We found that single *cp12-1*, *cp12-2*, and *cp12-3* mutants do not develop a severe photosynthetic or growth phenotype. In contrast, reductions of both *CP12-1* and *CP12-2* transcripts lead to reductions in photosynthetic capacity and to slower growth and reduced seed yield. No clear phenotype for *CP12-3* was evident. Additionally, the levels of PRK protein are reduced in the *cp12-1*, *cp12-1*/*2*, and multiple mutants. Our results suggest that there is functional redundancy between CP12-1 and CP12-2 in Arabidopsis where these proteins have a role in determining the level of PRK in mature leaves and hence photosynthetic capacity.

## Highlight

Reverse genetics approaches revealed functional redundancy between the CP12-1 and CP12-2 proteins and a role for these proteins in determining the level of the Calvin–Benson cycle enzyme phosphoribulokinase.

## Introduction

The Calvin–Benson cycle (CB) cycle is the primary pathway of carbon fixation producing carbon compounds for an array of connected pathways essential for plant growth and development. The functioning of this pathway is dependent on the availability of ATP and reducing power in the form of NADPH produced from photosynthetic electron transport. The supply of ATP and NADPH within the chloroplast varies in response to fluctuations in light intensity. One important mechanism that links the availability of products from the electron transport chain to the activity of the enzymes of the CB cycle is the ferredoxin/thioredoxin system (Buchanan, 1980; Scheibe, 1991; Geiger and Servaites, 1994; Schürmann and Jacquot, 2000; Schürmann and Buchanan, 2008; Michelet *et al.*, 2013). The activity of phosphoribulokinase (PRK) and glyceraldehyde-3-phosphate dehydrogenase (GAPDH), two enzymes of the CB cycle, is modulated by the ferredoxin/thioredoxin system. In the light, cysteine residues on thioredoxin are reduced, and this in turn brings about the reduction of cysteine residues on the PRK and GAPDH proteins, thereby activating these enzymes. In addition to the thioredoxin system. the activity of the enzymes PRK and GAPDH is further regulated by the formation of a multiprotein complex mediated by the small, nuclear-encoded chloroplast protein, CP12 (Pohlmeyer *et al.*, 1996; Wedel *et al.*, 1997; Scheibe *et al.*, 2002; Graciet *et al.*, 2004; Marri *et al.*, 2005*b*, 2009; Maberly *et al.*, 2010; Howard *et al.*, 2011*a*). The formation and breakdown of the PRK/GAPDH/CP12 complex *in vivo* has been shown to occur in response to changes in light availability, modulating the rapid deactivation and activation of PRK and GAPDH activity (Howard *et al.*, 2008).

The CP12 protein was first described in 1996 as a small protein with sequence similarity to the N-terminus of the B subunit of the CB cycle protein GAPDH, and it was shown that it was involved in the formation of a multiprotein complex made up of GAPDH, PRK, and CP12 (Pohlmeyer *et al.*, 1996; Wedel *et al.*, 1997; Wedel and Soll, 1998). Since then, a number of studies have described the mechanism by which the CP12 protein mediates the formation and breakdown of the PRK/GAPDH/CP12 complex *in vitro* (Graciet *et al.*, 2003*b*; Trost *et al.*, 2006; Marri et al., 2008, 2009, 2010; Erales *et al.*, 2009, 2011; Carmo-Silva *et al.*, 2011; Avilan *et al.*, 2012; Moparthi *et al.*, 2014, 2015). The CP12 proteins have a highly conserved primary structure, which includes three key features: an N-terminal cysteine pair, a C-terminal cysteine pair, and a core ‘AWD_VEE’ sequence. The N- and C-terminal cysteine pairs have been shown to form two intramolecular disulphide bridges when oxidized, which are converted to thiol groups when reduced by thioredoxin (Gardebien *et al.*, 2006; Groben *et al.*, 2010; Mileo *et al.*, 2013; Stanley *et al.*, 2013). Both the N- and C-terminal disulphide bridges are necessary for the formation of the GAPDH/CP12/PRK complex (Wedel and Soll, 1998; Graciet *et al.*, 2003*a*; Matsumura *et al.*, 2011; Del Giudice *et al.*, 2015). These proteins are universally distributed in oxygenic photosynthetic organisms, where at least one CP12-like protein has been identified in all species including cyanobacteria, with the exception of the prasinophyte *Osterococcus* (Wedel *et al.*, 1997; Wedel and Soll, 1998; Graciet *et al.*, 2003*a*; Marri *et al.*, 2005*a*; Tamoi *et al.*, 2005; Oesterhelt *et al.*, 2007; Robbens *et al.*, 2007; Groben *et al.*, 2010; Stanley *et al.*, 2013). Additionally, it has also been shown that proteins containing sequences with a high degree of similarity to the C-terminal region of CP12 are present in cyanophages (Thompson *et al.*, 2011). Interestingly, in the angiosperms, including *Oryza sativa*, *Arabidopsis thaliana*, *Solanum tuberosum*, *Brassica rapa*, and *Populus trichocarpa*, the CP12 proteins are encoded by a small gene family with up to three genes which can be grouped into two CP12 types (Singh *et al.*, 2008; Groben *et al.*, 2010). Cyanobacteria, on the other hand, have been shown to have highly diverse CP12 proteins which have been classified into eight different groups based on the presence or absence of the highly conserved features of the classical CP12 proteins, the N- and C-terminal cysteine pairs and the central highly conserved ‘AWD_VEE’ motif (Stanley *et al.*, 2013).

In the *A. thaliana* genome, three *CP12* genes have been identified and named *CP12-1* (At2g47400), *CP12-2* (At3g62410), and *CP12-3* (At1g76560). CP12-1 and CP12-2 proteins are highly similar, sharing up to 86% identity following cleavage of the transit peptide. Phylogenetic analyses of the *CP12* genes across a range of species have been unable to differentiate CP12-1 and CP12-2 into two separate subgroups on the basis of their amino acid sequence, and have been classified as CP12 type I. CP12-3 shares 47% and 46% identity with CP12-1 and CP12-2, respectively, and phylogenetic analysis places CP12-3 in a distinct clade as CP12 type II (Singh *et al.*, 2008; Groben *et al.*, 2010). Analysis of the expression patterns of the *CP12* gene family in Arabidopsis has shown that they are differentially expressed, raising questions about differential function of the individual CP12 proteins (Marri *et al.*, 2005*a*; Singh *et al.*, 2008). The expression of *CP12-2*, like *GAPDH* and *PRK*, is light dependent and is highest in photosynthetic tissues such as cotyledons, vegetative leaves, and stalks, although it is also present in some floral tissues. *CP12-1* transcripts, whilst abundantly expressed in photosynthetic tissues, are also evident in a wider range of tissues including flowers (siliques, styles, and sepals), seeds, and root tips. In contrast, *CP12-3* is expressed at lower levels and accumulates in roots, stigma, and anthers, but has very low expression in leaf tissue (Marri *et al.*, 2005*a*; Singh *et al.*, 2008). In addition, co-expression analysis of the Arabidopsis gene family has shown that the three *CP12* genes have very distinct co-expression patterns, suggesting that CP12 function may be important outside of the CB cycle (López-Calcagno *et al.*, 2014).

To date, only two studies have reported on the effects of reduced levels of CP12 protein *in vivo*, one using a cyanobacterial knockout mutant of *Synechococcus* PCC7942 (Tamoi *et al.*, 2005) and the other tobacco antisense CP12 plants (Howard *et al.*, 2011*a*, *c*). These studies indicate that CP12 has an important role in the regulation of metabolism, but potentially in different ways in the different organisms. In *Synechococcus* PCC7942, the results were consistent with the proposal that CP12 is necessary for the separation of the activity of the CB cycle from that of the oxidative pentose phosphate pathway during day–night cycles. In contrast, the results obtained from antisense CP12 tobacco plants showed a more complex phenotype. The reduced CP12 levels in the tobacco plants seemed to have a limited effect on the ability of the PRK/GAPDH/CP12 complex to form in the presence of NAD. Additionally, no significant differences in photosynthetic carbon fixation were detected. Furthermore, the CP12 antisense tobacco plants have a very slow growth rate and developed significant changes in morphology, including a loss of apical dominance, fused cotyledons, altered leaf shape, and reduced fertility. Changes in carbon allocation were also reported, with increased carbon being directed to the cell wall but with reduced carbon going to starch and soluble carbohydrates. Interestingly, the activity of the thioredoxin-activated enzyme NADP-malate dehydrogenase (NADP-MDH) was lower than in wild-type plants, and changes in pyridine nucleotide content were also evident. These results suggest a potential role for the CP12 protein(s) outside the formation of the regulatory PRK/GAPDH/CP12 complex (Howard *et al.*, 2011*a*). It has also been shown that the CP12-mediated regulation of PRK and GAPDH varies between different algal species (Maberly *et al.*, 2010) and that there is heterogeneity in the PRK and GAPDH protein complex in higher plant species. In *A. thaliana* it has not been possible to detect the *in vivo* presence of this complex (Howard *et al.*, 2011*b*). These results clearly suggest that the role of the CP12 proteins in higher plants is still not fully understood (Gontero and Maberly, 2012; López-Calcagno *et al.*, 2014).

The work presented herein aims to determine the importance of each of the CP12 family members *in vivo* in *A. thaliana*. To achieve this, Arabidopsis CP12 T-DNA mutants and plants expressing a *CP12*-targeted RNAi construct were used to study the importance of each individual gene in growth phenotype and photosynthetic performance.

## Materials and methods

### Plant growth conditions

Seeds of *A. thaliana* plants (Columbia-0 ecotype) were surface sterilized with 95% (v/v) ethanol+0.05% Tween for 5 min, and rinsed five times with 70% ethanol, allowed to dry, and then placed in Petri dishes containing half-strength Murashige and Skoog (1/2 MS) medium and 0.8% agar. Alternatively, seeds were put in sterile water. Freshly sown plates or seeds in water were stored at 4 °C and darkness for 48–72 h before moving to the light or sowing on soil. Pots or plates were then moved to a controlled environment chamber for seeds to germinate. All plants were grown in controlled conditions with either 16 h light/8 h dark (long days) or 8 h light/16 h dark (short days) and light levels of 130 μmol m^–2^ s^–1^.

### Identification and analysis of T-DNA CP12 mutants and production of multiple mutants

**Fig. 1.**
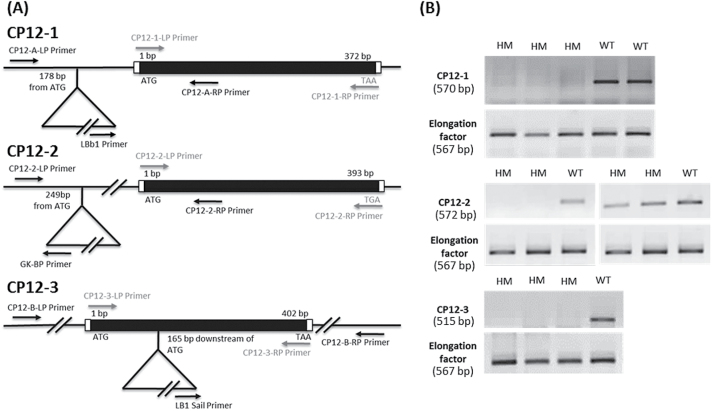
Molecular analysis of homozygous CP12 T-DNA insertion mutants. (A) Structure of the *CP12-1*, *-2*, and *-3* genes showing the location of T-DNA insertions. Protein-coding exons are represented in black, untranslated regions in white, and the location of genomic PCR primers are shown by black arrows. T-DNA insertion sites are indicated by triangles, and the precise position is given as the number of base pairs from ATG. ATG, translation initiation codon; TAA and TGA, translation termination codons. Locations of RT-PCR primers are shown by grey arrows. (B) Expression of the *CP12* genes in wild-type and mutant plants analysed by RT-PCR. Locations of the amplification primers are shown in (A). The elongation factor gene (AT1G07940.1) was used as a control. Amplicon sizes are indicated at the left under the gene name. For *CP12-1* and *CP12-3*, PCR amplifications were performed using 45 cycles. For *CP12-2*, the left gel shows PCR amplifications using 24 cycles and right gel using 25 cycles.

The *cp12-1*, *cp12-2*, and *cp12-3* mutants were identified in The Arabidopsis Information Resource (TAIR) database (lines SALK_008459.27.80.X, GK_397A01_017930, and SAIL_854_F09.v2, respectively). Mutant genotypes were assessed by PCR and the location of each T-DNA insertion was determined precisely (Fig. 1A) by sequencing junction-spanning PCR products (for primer details see Supplementary Table S1 at *JXB* online). Double mutants *cp12-1/2*, *cp12-1/3*, and *cp12-2/3* were obtained by crossing homozygous plants of each single mutant line and segregating the double homozygous plants. The triple mutant *cp12-1/2/3* line was similarly obtained by crossing double homozygous mutants and allowing its progeny to segregate until triple homozygous plants were identified.

### Quantitative real-time (qPCR) and standard RT-PCR

RNA was isolated using the nucleospin RNA/Protein kit from Macherey-Nagel (http://www.mn-net.com/). Reverse transcription was performed using RevertAid reverse transcriptase from Fermentas. For semi-quantitative analysis, RT-PCR products were resolved by electrophoresis and stained with ethidium bromide; amplification of a GTP-binding elongation factor gene (AT1G07940.1) was used as loading control. For qPCR, a minimum of three biological replicates were analysed, and each one was measured in triplicate using the iCycler iQ thermocycler (Bio-Rad) and SensiFast SYBR taq ready mix (Bioline). Data were normalized using similarly derived data for expression of: cyclophilin (AT2G29960), actin 2 (AT1G07940.1), and elongation factor (AT1G07940.1). Data analysis was done using the Bio-Rad CFX Manager 3.1.

### Building construct for complementation

The sequence of the *CP12-1* gene of *A. thaliana* including the promoter and coding sequence (CDS) was retrieved from the TAIR database, the promoter area was selected as described previously (Singh *et al.*, 2008), and primers were designed accordingly (see details in Supplementary Table S1). The promoter–CDS fragment was amplified as a single fragment from genomic DNA and the construct was prepared using Gateway technology (Invitrogen). The target sequence was first captured in a commercially available pENTRY/D-TOPO vector (Invitrogen) and then recombined with the plant destination vector pEarleyGate 302 (Earley *et al.*, 2006) for expression. The sequences of the cloning and expression vectors were confirmed by sequencing. The construct was stably introduced into *cp12-1/2/3* mutant plants using the floral dip method (Clough and Bent, 1998). Transformants were screened by germination and early development on soil moistened with 0.62 mM of glufosinate-ammonium (Kaspar, from Bayer crop Science). Growth comparisons were done in T_2_ and T_3_ generations.

### Building the *CP12-2* RNAi construct

For further reductions of *CP12-2* expression, two RNAi constructs were built using Golden Gate cloning (Engler *et al.*, 2008, 2009). RNAi constructs contained two copies of either a 126 bp (construct 53) or a 179 bp (construct 54) region of the *CP12-2* gene in opposite orientations separated by intron 8 (236 bp) from the Arabidopsis plastid terminal oxidase (PTOX) (EMBL accession no. ATT10I14). These were then assembled together with a *Cauliflower mosaic virus* 35S promoter and a NOS terminator into a plant expression construct using Golden Gate technology. Arabidopsis plants were transformed using the floral dip method (Clough and Bent, 1998) and transformants were identified by screening on 1/2 MS medium agar plates containing 10 μg ml^–1^ hygromycin.

### Gas exchange analysis

The response of net photosynthesis (*A*) to substomatal CO_2_ concentration (*C*_i_) was measured using a portable gas exchange system (CIRAS-1, PP Systems Ltd, UK). The measurement procedure was done as described in Simkin *et al.* (2017) with some modifications. Leaves were illuminated with an integral red–blue LED light source (PP Systems Ltd) attached to the gas exchange system, and light levels were maintained at a saturating photosynthetic photon flux density (PPFD) of 1000 μmol m^–2^ s^–1^ for the duration of the *A/C*_i_ response curve. Measurements of *A* were made at ambient CO_2_ concentration (*C*_a_) at 400 μmol mol^–1^, before *C*_a_ was decreased to 300, 200, 100, and 50 μmol mol^–1^. Following this, the *C*_a_ was increased stepwise to 1500 μmol mol^−1^ for completion of the curve in nine steps (150, 250, 350, 450, 550, 700, 900, 1100, and 1500 μmol mol^−1^). The CO_2_ assimilation rate per unit leaf area and intercellular CO_2_ concentration (*C*_i_) were determined using the equations of von Caemmerer and Farquhar (1981), and were used to calculate the maximum rates of ribulose-1,5-bisphosphate carboxylase/oxygenase (Rubisco; *V*_cmax_) and the maximum rate of electron transport for ribulose bisphosphate (RuBP) regeneration (*J*_max_) to a leaf temperature of 25 °C based on equations from Sharkey *et al.* (2007).

The response of net photosynthesis to changes in light was also measured both as light dose–response curves (*A*/*Q*) and as transients; all these were measured at ambient CO_2_ concentration (400 μmol mol^–1^). For *A*/*Q* curves, leaves were initially stabilized at saturating irradiance 1000 μmol m^–2^ s^–1^, after which *A* and stomatal conductance (*g*_s_) were measured at light levels between 0 to 1000 μmol m^–2^ s^–1^. Measurements were recorded after *A* reached a new steady state (1–2 min) and before *g*_s_ changed to the new light levels.

For transients, CO_2_ assimilation was measured every 30 s following a step change in light intensity. Prior to measurements, plants were acclimated to dark or low light (41 μmol m^–2^ s^–1^), after which light was increased to 500 μmol m^–2^ s^–1^. After 10–15 min at the higher light intensity, light was returned to the original value.

### Chl *a* fluorescence imaging

Images of Chl *a* fluorescence were obtained as described by Barbagallo *et al.* (2003) using a CF Imager (Technologica Ltd, Colchester, UK).

Seedlings at 14, 18, 20, and 22 days after sowing (DAS) grown in a controlled environment chamber at 130 μmol m^–2^ s^–1^ and ambient (400 μmol m^–1^ s^–1^) CO_2_ were imaged directly in 1/2 MS agar plates. The operating efficiency of PSII photochemistry, *F*_q_′/*F*_m_′, was calculated from measurements of steady-state fluorescence in the light (*F*’) and maximum fluorescence in the light (*F*_m_′) which were obtained after a saturating 800 ms pulse of 6200 μmol m^–2^ s^–1^ PPFD using the following equation *F*_q_′/*F*_m_′=(*F*_m_′–*F*′)/*F*_m_′. Images of *F*_q_′/*F*_m_′ were taken under a stable PPFD of 130 μmol m^–2^ s^–1^ and a PPFD of 300 μmol m^–2^ s^–1^. The significance of data obtained was statistically tested by running an ANOVA followed by Tukey test using R (https://www.r-project.org/).

The response of *F*_q_′/*F*_m_′ to changes in light intensity was also monitored. For this, mature rosettes were imaged. Prior to measurements, pre-dawn or 20 min dark-adapted plants were used to obtain the minimal fluorescence (*F*_o_), which was measured using a weak measuring pulse. Maximal fluorescence (*F*_m_) was measured using an 800 ms long saturating light pulse of 6200 μmol photons m^–2^ s^–1^. Plants were then exposed to an actinic light of 50 μmol m^–2^ s^–1^ PPFD for 20 min, and steady-state *F*’ was continuously monitored, while maximum fluorescence in the light (*F*_m_’) was measured at 1 min intervals by applying saturating light pulses; light was then increased to 400 μmol m^–2^ s^–1^ PPFD for 10 min, and then returned to 50 μmol m^–2^ s^–1^ PPFD for 15 min. Fluorescence was monitored every minute as described. Using the images captured at *F*′ and *F*_m_′, images of operating efficiency of PSII (*F*_q_′/*F*_m_′) were constructed by the imaging software (FluorImager).

### Growth analysis (leaf and rosette area calculations)

For total leaf area and rosette area calculations, pictures of the seedlings in agar and older plants on soil were taken and processed using ImageJ (http://rsb.info.nih.gov/ij/index.html). Within the plates, the 10 larger plants of each plate (containing up to 50 seedlings) were registered, while all the plants grown in soil were measured. Alternatively, in plants where chlorophyll fluorescence measurements were done using the CF Imager, the software used for calculation of the fluorescence parameters also was used to calculate total leaf/rosette area. The significance of data obtained was statistically tested by Kruskal–Wallis analysis followed by Man–Whitney tests (*P*<0.05) using SPSS (Statistical Product and Service Solutions) from IBM or by running an ANOVA followed by Tukey test using R (https://www.r-project.org/)

### Total protein extraction and immunoblotting protocol

Frozen leaf tissue (30 mg) was ground to a powder using liquid nitrogen and a cold mortar and pestle. Protein extraction buffer [50 mM HEPES pH 8.2, 5 mM MgCl_2_, 1 mM EDTA, 10% glycerol, 0.1% Triton X-100, 2 mM benzamidine, 2 mM aminocaproic acid, 0.5 mM phenylmethanesulphonyl fluoride (PMSF), and 10 mM DTT] was added and ground further before transferring to a cold 1.5 ml microcentrifuge tube. Insoluble material was removed by centrifugation at 14 000 *g* for 2 min (4 °C). The supernatant was collected and aliquoted for protein quantification (Bradford assay) and further analysis. The presence of the CP12-FLAG protein was assessed in these extracts by immunoblots, employed as previously described (Kovacheva *et al.*, 2005), using anti-rabbit IgG (H+L) horseradish peroxidase- (HRP) conjugated secondary antibodies (Promega), and the detection reagent Pierce ECL Western Blotting Substrate (Thermo Scientific). The following antibodies were used: sedoheptulose-1,7-bisphosphatase (SBPase; Lefebvre *et al.*, 2005), fructose-1,6-bisphosphate aldolase (FBPA; Simkin *et al.*, 2015), transketolase (TK; Henkes *et al.*, 2001), Rubisco (Foyer *et al.*, 1993), phosphoribulokinase (PRK; Klein and Salvucci, 1995), GAPDH (Pohlmeyer *et al.*, 1996), malate dehydrogenase (MDH; Fickenscher and Scheibe, 1983), and NADPH-thioredoxin reductase C (NTRC; Lepistö *et al.*, 2009). In addition to the aforementioned antibodies, samples were probed using antibodies raised against the small subunit of ADP-glucose pyrophosphorylase (ssAGPase; AS111739), the PSI type I Chl *a*/*b*-binding protein (Lhca1; AS01005), ATPδ (AS101591), Rieske FeS (PetC: AS08330), and against the glycine decarboxylase H-subunit (AS05074), purchased from Agrisera (via Newmarket Scientific UK).

For investigation of protein redox states, proteins were extracted using trichloroacetic acid (TCA); leaf tissue (100–50 mg) was flash-frozen in liquid nitrogen and ground in an eppendorf tube on dry ice, TCA (500 μl, 10% w/v) was added, and incubated on ice for 10–20 min. Samples were spun at 14 000 *g* and 4 °C for 15 min, and washed twice with ice-cold acetone (80% v/v) followed by 100% ice-cold acetone. Samples were then vacuum dried and proteins resuspended in 150 μl of Resuspension Buffer [62.5 mM Tris–HCl (pH 7.5), 2% (w/v) SDS, 7.5% (v/v) glycerol, 0.01% (w/v) bromophenol blue, and Sigma’s protease inhibitor cocktail]. Samples were then treated with H_2_O_2_, with DTT, or kept in ice prior to 10 mM 4-acetamido-4′-maleimidylstilbene-2,2′-disulphonic acid (AMS) treatment. AMS treatment was performed overnight at 4 °C, and samples were then separated by non-reducing SDS–PAGE.

### Accession numbers

The gene sequences mentioned in this study can be found in the Arabidopsis Genome Initiative database under the following accession numbers: AT2G47400 (*CP12-1*), AT3G62410 (*CP12-2*), AT1G76560 (*CP12-3*), AT3G18780 (*Actin2*), AT1G07940 (*EF*), AT2G29960 (*Cyclophilin*), and AT1G32060 (*PRK*).

## Results

### Identification and analysis of Arabidopsis CP12 T-DNA insertion mutants

To elucidate the functional significance of the individual members of the Arabidopsis CP12 gene family *in vivo*, we identified one T-DNA insertion mutant for each of the three genes in the TAIR database (http://www.arabidopsis.org/). A wide collection of CP12 polymorphisms were found (66), 23 of which are insertion mutants (11 lines for CP12-1, 2 lines for CP12-2, and 10 lines for CP12-3). The three best candidate lines, SALK_008459.27.80.X (*cp12-1*), GK_397A01_017930 (*cp12-2*), and SAIL_854_F09.v2 (*cp12-3*), were selected based on the position of the insertion of the T-DNA to enable disruption of the exon or hit the promoter to interfere with gene expression. All T-DNA insertion sites were confirmed using PCR analysis (data not shown) of genomic DNA followed by sequencing of the T-DNA–gene junctions. Diagrams of the positions of the T-DNA inserts are presented in Fig. 1A.

Homozygous plants (identified by PCR analysis) for each of the lines were used to assess the effect of each T-DNA insertion on *CP12* gene expression by qPCR (Fig. 1B). Semi-quantitative RT-PCR analysis confirmed that the relevant full-length mRNA was absent for *cp12-1* and *cp12-3* T-DNA lines, and qPCR found no amplification of the selected amplicons, providing evidence that the lines are true knockout (KO) alleles. In contrast detectable levels of the *CP12-2* transcript were found in the *cp12-2* T-DNA line, and so this is considered to be a knockdown (KD) allele. Preliminary observations of these three mutant lines failed to reveal any major growth or developmental phenotypes. To investigate further the impact of the lack or decreased levels of the *CP12* gene transcripts, double and triple mutant lines were generated. It was not possible to determine changes in protein levels as detection of CP12 protein in leaf extracts has not proved possible.

### Production and analysis of CP12 single, double, and triple mutants

**Fig. 2.**
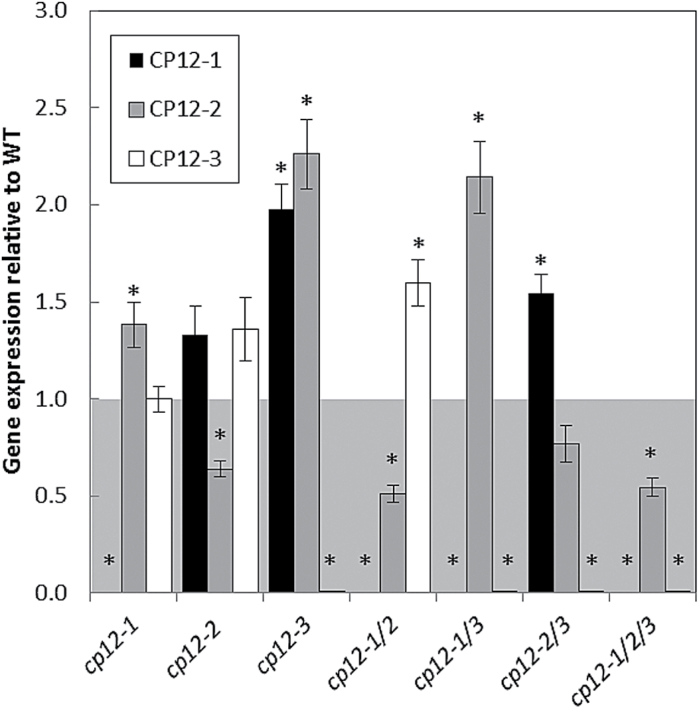
qRT-PCR analysis of *CP12* gene expression in the T-DNA mutants. Relative levels of expression of the CP12 genes in the CP12-T-DNA mutant collection with respect to wild-type (WT) *CP12* expression levels. Mean values±SE are indicated, *n*=3–4 biological replicates. *Actin2* (AT3G18780), the elongation factor gene (AT1G07940.1), and *Cyclophilin* (AT2G29960) were used as internal standards (nean CV, 0.1398; mean M value, 0.3534). An asterisk indicates significant differences between the WT and mutant level of expression (*P*<0.05).

The double mutant lines *cp12-1/2*, *cp12-1/3*, and *cp12-2/3*, and the triple mutant line *cp12-1.1/2.1/3.1* were identified using PCR analysis of genomic DNA (Supplementary Fig. S1). The impact of the single and multiple T-DNA insertions on the expression of the *CP12* genes was evaluated using qPCR analysis (for primer details, see Supplementary Table S1). The results, presented in Fig. 2, show clearly that the insertions in *CP12-1* and in *CP12-3* yield KO mutants, as no expression of these genes is detectable in either the single or multiple mutants. In contrast, RNA transcripts for *CP12-2* were detected in the insertion mutant for the *CP12-2* gene, indicating that this is a KD mutant with 51–77% of the wild-type RNA levels. This analysis also showed that the T-DNA KO and KD effect was observed in the double and triple mutants.

**Fig. 3.**
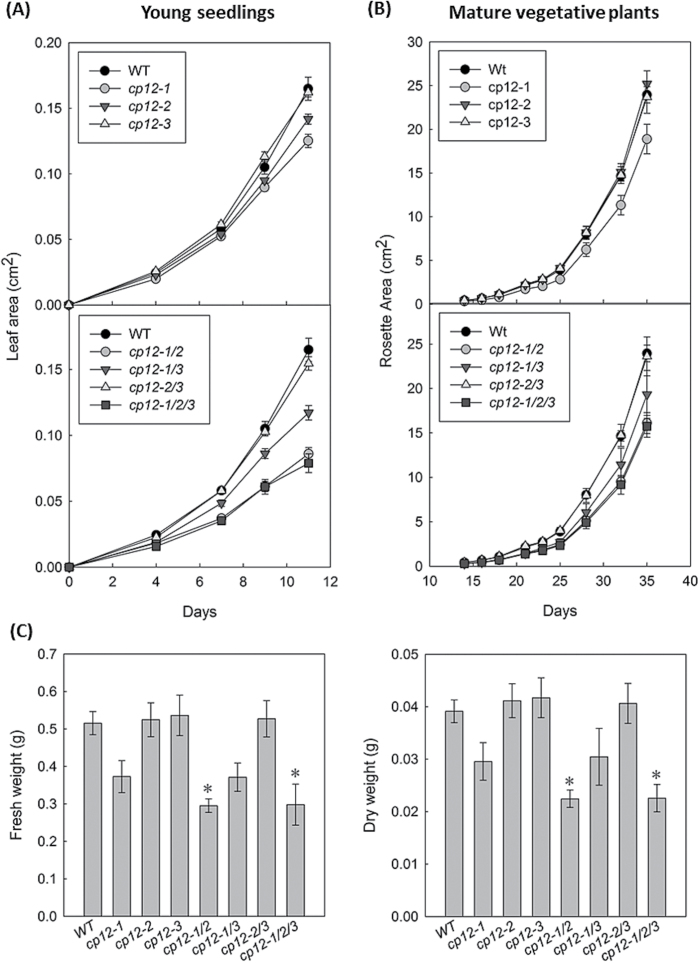
Growth analysis of wild-type (WT) and CP12 T-DNA insertion mutant plants. Plants were grown for 7 weeks in a controlled environment growth room (22 °C, 8 h light, 16 h dark cycle). *Arabidopsis thaliana* (Col-0) WT, *cp12-1*, *cp12-2*, *cp12-3*, *cp12-1/2*, *cp12-1/3*, *cp12-2/3*, and *cp12-1/2/3* mutants are shown. (A) Seeds were germinated on 1/2 MS plates and seedlings grown for 14 d. Rosette area was recorded every 2–3 d. (B) Plants were germinated and grown on soil and the rosette area recorded from 14 to 35 DAS. (C) Fresh and dry weights of rosettes harvested at 36 DAS. Mean values±SE are indicated in all graphs, *n*=8–10. In (C), asterisks indicate significant differences between the WT and mutants (*P*<0.05).

In order to evaluate the impact of these changes on the transcription levels of the CP12 family on plant development, three independent growth experiments were performed on the CP12 mutant collection. Seeds from all mutant genotypes were germinated and grown on agar plates (1/2 MS with no sucrose). Leaf area was recorded every 2–3 d between days 4 and 11, and this showed a slow growth phenotype in the *cp12-1/2/3* triple mutant and the *cp12-1/2* double mutant, which achieved only 48% and 52% of the total leaf area of wild-type seedlings by day 11, respectively (Fig. 3A). Small decreases in leaf area were also observed in *cp12-1*, *cp12-2*, and *cp12-1/3* mutants (Fig. 3A). A second batch of plants was germinated and grown on soil, and used to investigate changes in growth at later stages of development. Again a slow growth phenotype was evident, with *cp12-1/2* and *cp12-1/2/3* achieving only 67%, and 66% of wild-type rosette area, respectively, after 5 weeks of growth (Fig. 3B). In this experiment, the *cp12-1* and *cp12-1/3* mutants also showed slow growth, achieving only 79% and 81% of wild-type area, respectively, after 5 weeks of growth. In contrast, *cp12-2*, *cp12-3*, and *cp12-2/3* had a similar growth to that of wild-type plants. These growth responses are also seen in leaf number (Supplementary Fig. S2) and in fresh and dry weight of the rosettes after 36 d growth (Fig. 3C).

Growth of the CP12 mutants in short days showed that the *cp12-1/2/3* and the *cp12-1/2* mutants produced less seed than the wild type in two independent experiments done 2 years apart; yields for *cp12-1/2/3* mutants were found to be between 48% and 71% of wild-type yield (Supplementary Fig. S3). Interestingly, individual seeds of the *cp12-1/2/3*, *cp12-1/2*, and *cp12-1* plants weighed significantly less than those of the wild type, and the *cp12-1/2/3* seeds were 28% below the weight of wild-type seed.

### Evaluation of the relative importance of CP12-1 and CP12-2

The results presented here so far suggest that CP12-1 makes a major contribution to the development of the growth phenotype described. To explore this further, we expressed a CP12-1-FLAG protein in the triple mutant.

For this, a construct for expression of a Flag-tagged CP12-1 protein, driven by the native promoter for this gene (Singh *et al.*, 2008) was produced (Supplementary Fig. S4A). Triple mutant plants were transformed and four independent lines identified. These transgenic lines were analysed to confirm the presence of the construct and production of the Flag-tagged protein and, based on these analysis, two independent lines (Supplementary Fig. S5) were used to evaluate the recovery of the slow growth phenotype. Supplementary Fig. S4B and C show how the triple mutant slow growth phenotype was complemented by the expression of a CP12-1-FLAG protein. The rosette area of these transgenic lines was analysed 22 and 29 DAS, showing no significant differences tfrom the wild type and always being statistically significantly larger than the *cp12-1/2/3* plants.

**Fig. 4.**
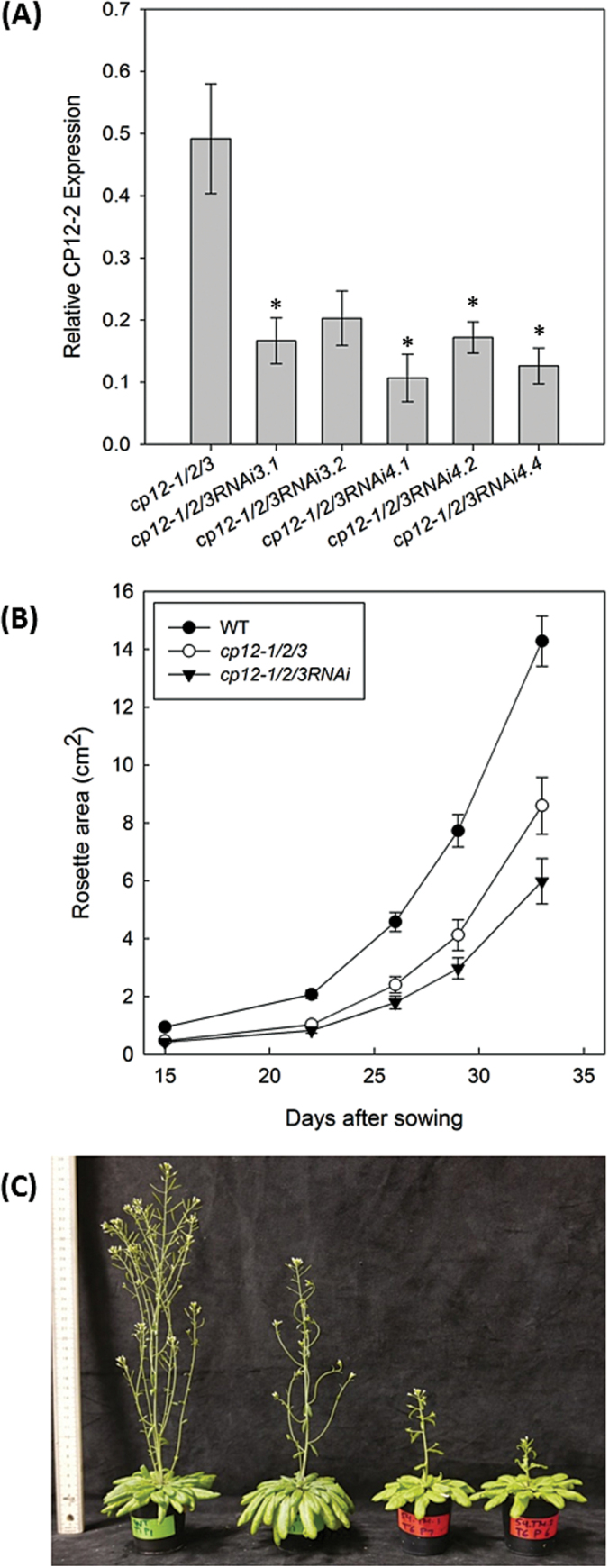
RNAi down-regulation of CP12-2 expression results in further reductions in growth on the *cp12-1/2/3* mutant. (A) Expression of the *CP12-2* gene in *cp12-1/2/3RNAi* plants. Quantitative RT-PCR analysis of total RNA from leaf tissue from 47-day-old plants used in the growth experiment. The results are the mean from 3–6 biological replicates, and the error bars indicate the SE. *Actin2* (AT3G18780) and *Cyclophilin* (AT2G29960) were used as internal standards (mean CV, 0.2368; mean M value, 0.6806). An asterisk indicates a significant difference between the wild-type (WT) and mutant level of expression (*P*<0.05). (B) Analysis of WT and mutant Arabidopsis plants grown in soil under controlled environment conditions of 8 h light/16 h dark at 22 °C, light level of 130 μmol m^–2^ s^–1^. Each group is represented by 14–15 plants. The Cp12-1/2/3RNAi group represents five independent lines pooled together (no statistically significant differences were detected between these lines). Mean values±SE are indicated. (C) Increased severity of growth phenotype evident in mature flowering plants (77 DAS).

Given that the CP12-2 T-DNA mutant line used in this study is a KD, and not a KO, an RNAi approach was taken to reduce the *CP12-2* transcript levels further, enabling us to address more fully the role of CP12-2. RNAi constructs (Supplementary Fig. S6) were transformed into *cp12-1/2/3* plants with the aim of obtaining triple KO mutants. Seven independent transgenic lines were produced and *CP12-2* expression was evaluated by qPCR. Five lines were selected based on the most severe reductions of the *CP12-2* transcript and used for further analysis (data not shown). Growth analysis was undertaken as described before in Fig. 3, for wild-type, *cp12-1/2/3*, and *cp12-1/2/3RNAi* lines. These experiments showed that the *cp12-1/2/3RNAi* plants with further decreased *CP12-2* transcript (Fig. 4A) showed a more dramatic growth phenotype. During the vegetative stage of growth. the rosette areas were smaller than in the *cp12-1/2/3* mutants, flowering was delayed, and seed yield was also reduced when compared with that of the *cp12-1/2/3* triple insertion mutant (Fig. 4B, C; Supplementary Fig. S3). Additionally, measurements of specific leaf area (SLA) showed increases in SLA in *cp12-1/2/3*, and revealed statistically significant increases in the SLA of *cp12-1/2/3RNAi* plants when compared with wild-type plants (Supplementary Fig. S7).

Due to the severity of the cp12-1/2/3RNAi phenotype (low seed yields, germination, and reduced growth), we focused the rest of the work on two independent lines with intermediate phenotypes: *cp12-1/2/3RNAi3.1*, referred to as *RNAi3*; and *cp12-1/2/3RNAi4.2*, referred to as *RNAi4.* To investigate further the slow growth phenotype of these plants, we looked at root development. Seedlings were grown on vertical plates for 24 d and roots scanned every 3 d to monitor root length, number of lateral roots, and the ratio between the number of lateral roots and the primary root length in wild-type, *cp12-1/2/3*, and *cp12-1/2/3RNAi* plants. Root length was reduced in the CP12 mutants and, somewhat more interestingly, the ratio between the number of lateral roots and the primary root length showed that in both *cp12-1/2/3* and the *cp12-1/2/3RNAi* plants the number of lateral roots is reduced relative to wild-type plants (Supplementary Fig. S8).

### Decreased levels of *CP12* transcripts leads to reduced PRK protein levels

**Fig. 5.**
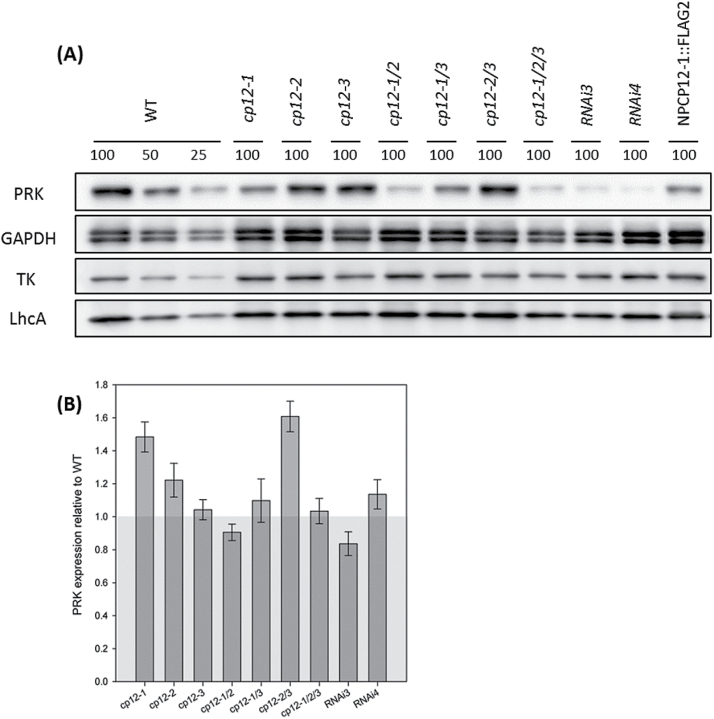
Lack of CP12-1 leads to decreased levels of PRK protein. (A) Equal crude protein samples from mature rosettes of the indicated genotypes were analysed by immunoblotting using the antibodies shown. PRK (phosphoribulokinase), GAPDH (glyceraldehyde-3-phosphate dehydrogenase), TK (transketolase), and LhcA (light-harvesting complex). Each sample represents a pool of 2–4 different plants. (B) qRT-PCR analysis of PRK gene expression in CP12 mutants. Relative levels of expression of the PRK gene in the CP12-T-DNA mutant collection and *cp12-1/2/3RNAi* plants with respect to wild-type (WT) expression levels. The results are the mean from 3–4 biological replicates, and the error bars indicate the SE. *Actin2* (AT3G18780), the elongation factor gene (AT1G07940.1), and *Cyclophilin* (AT2G29960) were used as internal standards (mean CV, 0.1869; mean M value, 0.4668).

To explore the underlying causes of the growth and development phenotypes described, the relative amounts of PRK and GAPDH and other chloroplast proteins were determined using immunoblot analysis. No differences were found between wild-type, *cp12-1/2/3*, and *cp12-1/2/3/RNAi* plants for CB cycle proteins GAPDH, FBPA, SBPase, TK, or Rubisco, for other chloroplast proteins, MDH, Lhca1, Rieske FeS, ATPδ, or ssAGPase, or even for the mitochondrial H-protein from the glycine decarboxylase complex involved in photorespiration. In contrast, PRK levels were reduced substantially in all of the mutants lacking the *CP12-1* transcript, with particularly high reductions of >85% of wild-type PRK protein levels in the *cp12-1/2/3* and the *cp12-1/2/3RNAi* plants (Fig. 5A; Supplementary Figs S9, S10). Analysis of the PRK transcript levels using qRT-PCR showed no decrease between the mutants and wild type (Fig. 5B). In the mutant lines complemented with the NPCP12-1::FLAG, the levels of PRK are equivalent to or higher than those observed in the *cp12-1/2/3* mutant.

To explore the possibility that the decrease in PRK levels was caused by changes in the redox state of the PRK protein, non-reducing SDS–PAGE followed by immunoblotting was performed on wild-type, *cp12-1/2/3*, and *cp12-1/2/3RNAi* leaf tissue collected in the middle of the light period. No detectable differences in the redox state of PRK or TK were detected between wild-type and CP12 mutant plants at this point in development (Supplementary Fig. S11).

### Photosynthetic rates in mature plants are only affected in CP12 mutants with extreme reductions in PRK protein

**Fig. 6.**
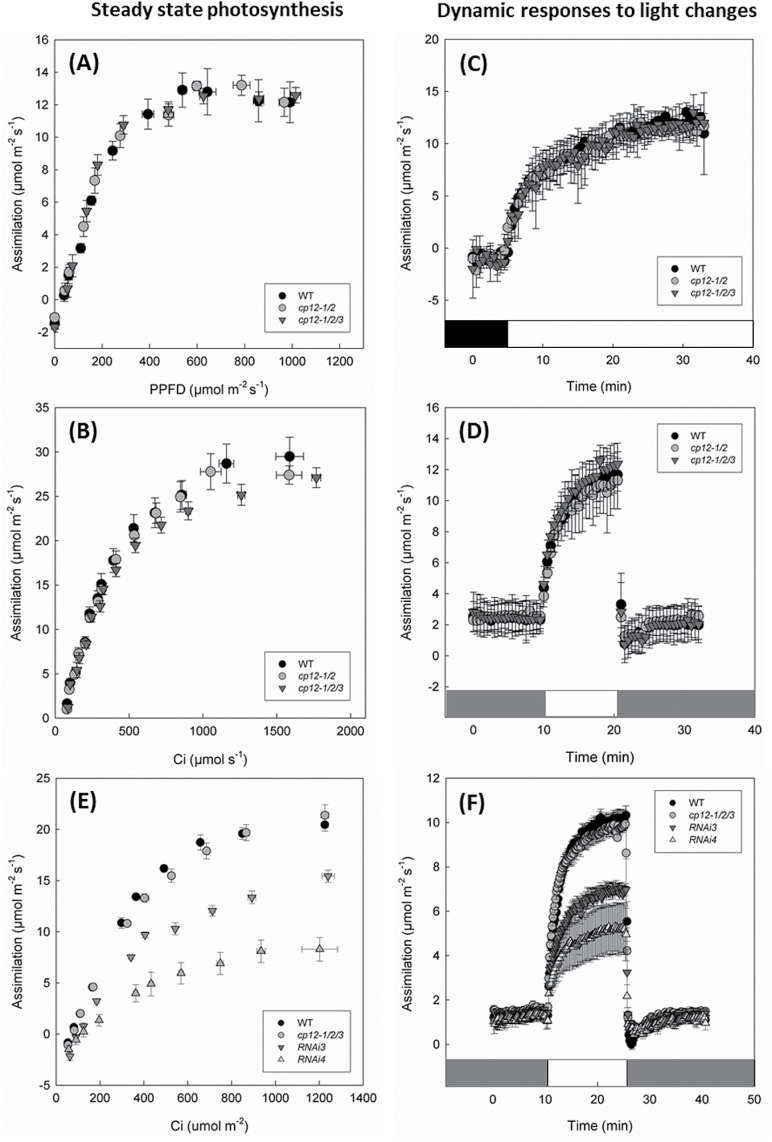
Photosynthetic carbon assimilation rates in *cp12-1/2*, *cp12-1/2/3*, and *cp12-1/2/3RNAi* Arabidopsis mutants. (A) Over a range of light intensities (PPFD, photosynthetic photon flux density). (B) In response to a range of CO_2_ concentrations. (C) In plants dark adapted for 20 min followed by 500 μmol m^–2^ s^–1^ light. (D) In plants adapted to low light (60 μmol m^–2^ s^–1^) for 20 min followed by ~500 μmol m^–2^ s^–1^ for 10 min and then returned to low light. Data are the means of 4–8 replicates (±SE). (E) Photosynthetic CO_2_ assimilation in response to a range of CO_2_ concentrations. (F) In plants adapted to low light (41 μmol m^–2^ s^–1^) for 20 min followed by ~500 μmol m^–2^ s^–1^ for 15 min and then returned to low light. For (E) and (F), data are the means of 4–5 replicates (±SE).

Given the slow growth phenotype described and the known role of the CP12-1 and CP12-2 proteins in the regulation of the CB cycle enzymes PRK and GAPDH, the rates of photosynthetic carbon assimilation were assessed in the two T-DNA lines with the most severe phenotypes (*cp12-1/2* and *cp12-1/2/3*) and the wild type. Assimilation rates (*A*) as a function of light intensity (*A*/*Q*) and intercellular CO_2_ [net CO_2_ assimilation rate (*A*) versus calculated substomatal CO_2_ concentration (*C*_i_) response curve] were determined. The light dose–response curves measured in the *cp12-1/2* and *cp12-1/2/3* mutants showed no significant differences when compared with wild-type plants. (Fig. 6A). Analysis of the *A/C*_i_ response showed that under light- and CO_2_-saturated conditions, the average values obtained for the *cp12-1/2/3* plants were lower than for either thhe wild type or *cp12-1/2*, indicating some impact on photosynthesis; however, these values were not found to be statistically different (Fig. 6B). Furthermore, the maximum carboxylation rates of Rubisco (*V*_cmax_) and the maximum rate of electron transport for RuBP regeneration (*J*_max_) showed no significant differences between the mutants and wild type (data not shown). Considering that the GAPDH/CP12/PRK complex has been described as sensitive to dynamic light (Howard *et al.*, 2008), it seems plausible that differences in assimilation would appear immediately after a change in irradiance and that these may disappear at steady state. Based on this, photosynthesis was determined when plants were subjected to a step increase in light, from total darkness or low light (near 50 μmol m^–2^ s^–1^) to light intensities of 500 μmol m^–2^ s^–1^; however, even under these conditions, no significant differences in assimilation rates were found (Fig. 6C, D). To explore further the photosynthetic performance of the CP12 mutants, chlorophyll fluorescence imaging was used to assess the operating efficiency of PSII photochemistry (*F*_q_’/*F*_m_’); no significant differences were found in plants 4–6 weeks after planting (Supplementary Fig. S12).

In light of these results and considering that in tobacco PRK antisense plants, reductions in photosynthesis were only evident in plants with ≤20% of wild-type PRK levels (Paul *et al.*, 1995; Habash *et al.*, 1996), CO_2_ assimilation and PSII operating efficiency were evaluated in mature *cp12-1/2/3RNAi* plants. Figure 6E and F shows how the further decrease in the *CP12-2* transcript and consequent decrease in PRK protein results in a significant decrease in photosynthesis, as a function of both CO_2_ concentration and photosynthetic efficiency following a transient increase in light intensity. The *A*/*C*_i_ curve analysis revealed significant decreases in *V*_cmax_ and *J*_max_ in the *RNAi* plants when compared with the *cp12-1/2/3* mutants and wild-type plants (Table 1). This decrease in photosynthesis was also observed when plants were subjected to a step increase in light, from low light (50 μmol m^–2^ s^–1^), to light intensities of 500 μmol m^–2^ s^1^. Although no clear differences in CO_2_ assimilation were evident at low light intensities, once the plants were exposed to higher light, the assimilation rates of the *cp12-1/2/3RNAi* plants were much lower than those of the wild type and *cp12-1/2/3* mutants. Line *RNAi4*, which revealed the lowest levels of PRK protein in immunoblots, also displayed the most severe impact on photosynthesis (Fig. 6E, F). Additionally, *F*_q_′/*F*_m_′ was also significantly lower in the *RNAi* plants throughout the transient, whilst *F*_q_′/*F*_m_′ was only lower than that in the wild type at the higher light level (500 μmol m^–2^ s^–1^) in the *cp12-1/2/3* mutants (Supplementary Fig. S13). It should also be noted that the dark-adapted maximum quantum efficiency of PSII photochemistry (*F*_v_/*F*_m_) was significantly lower in the *RNAi* lines, suggesting reduced efficiency or damage to the photosystems.

**Table 1.** *V*_cmax_ and *J*_max_ values (μmol m^–2^ s^–1^) for wild-type (WT), *cp12-1/2/3*, and *cp12-1/2/3RNAi* plants calculated from the *A*/*C*_i_ curves presented in Fig. 6E

	*V*_cmax_±SD (at 25 °C)	*J*_max_±SD (at 25 °C)
WT	89.4 ± 15.0	149.0 ± 14.9
*cp12-1/2/3*	87.6 ± 16.5	142.1 ± 17.5
*RNAi3*	51.3 ± 16.8	99.1 ± 18.1
*RNAi4*	37.9 ± 13.0	70.5 ± 18.6

**Fig. 7.**
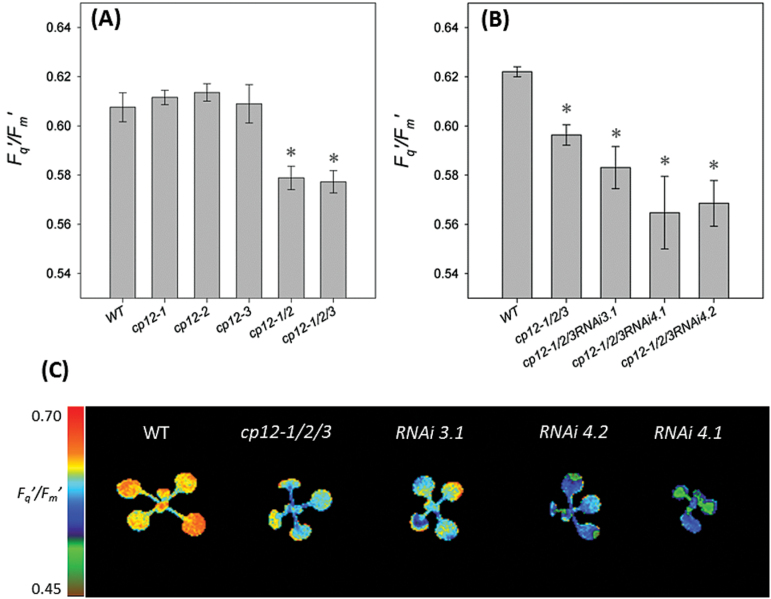
Lack of CP12-2 can further reduce photosynthetic efficiency in young seedlings. Determination of photosynthetic efficiency in wild-type (WT), *cp12-1/2/3*, and *cp12-1/2/3RNAi* seedlings using chlorophyll fluorescence imaging. WT and mutant plants were grown in controlled environment conditions with a light intensity of 130 μmol m^–2^ s^–1^; *F*_q_′/*F*_m_′ (maximum PSII operating efficiency) was determined at 130 μmol m^–2^ s^–1^ light intensities. (A) T-DNA mutant collection. (B and C) WT, *cp12-1/2/3*, and RNAi transformants. The scale bar represents an *F*_q_′/*F*_m_′ of 0.45–0.70. The data were obtained using 5–10 individual plants from each line. Mean values±SE are indicated. An asterisk indicates values significantly lower than the WT (*P*<0.05).

Although no differences in photosynthesis were evident in mature plants of the T-DNA mutant lines, some of these mutants still present a severe slow growth phenotype (*cp12-1/2* and *cp12-1/2/3* lines). Since this phenotype is evident during the early stages of vegetative growth, we assessed the photosynthetic capacity of the *cp12-1/2*, *cp12-1/2/3*, and *cp12-1/2/3RNAi* lines compared with wild-type and single T-DNA mutant plants in early stages of development using chlorophyll fluorescence imaging. *F*_q_′/*F*_m_′ was determined in young seedlings (15–22 DAS) grown in 1/2 MS agar, under a light intensity similar to growth light (130 μmol m^–2^ s^–1^). No differences were evident between the wild type, *cp12-1*, *cp12-2*, and *cp12-3* single mutants; but statistically significant lower values for *F*_q_′/*F*_m_′ were found in *cp12-1/2*, *cp12-1/2/3*, and *cp12-1/2/3RNAi* plants when compared with the wild type (Fig. 7).

## Discussion

CP12 T-DNA insertion lines with the potential to disrupt the expression of each member of the *CP12* gene family were identified in *A. thaliana* and, together with lines expressing an RNAi constructs to knock down *CP12-2* further, were used to evaluate the functional importance of the *CP12* gene family in plant growth and development. The results presented here show that absence or reduction of the expression of two or more *CP12* genes generates a slow growth phenotype in *A. thaliana* under normal growth conditions. The need for more than one gene transcript to be reduced for the development of the phenotype suggests a certain level of functional redundancy between family members. Furthermore, the severity of the phenotypes developed suggests that CP12-1 and CP12-2 proteins have the dominant roles in the family. No changes in growth were detected in the *cp12-3* mutants nor was the phenotype of the multiple mutants containing a KO *CP12-3* gene increased in severity.

The development of the most severe phenotypes in the lines with insertions in two or more genes (particularly *cp12-1/2*, *cp12-1/2/3*, and *cp12-1/2/3RNAi*) suggested that there is some functional redundancy between members of the CP12 family. Additionally, the up-regulation of the *CP12* transcripts in the T-DNA mutants when another member of the family was knocked out or knocked down by a T-DNA insert (as shown in Fig. 2) supports the assumed redundancy in the case of the CP12 family in Arabidopsis. This is a common phenomenon in plants, and has been found numerous times with gene families involved in a range of diverse functions including nitrogen transporters (Kaiser *et al.*, 2002), protein kinases (Fujii *et al.*, 2011), and the heavy metal-transporting ATPase (Mills *et al.*, 2010). In general, this phenomenon is found when there is a high similarity between family members, usually due to being products of gene duplications, and when these gene products are capable of performing the same function at the same place and time (Kafri *et al.*, 2005, 2006). Some of these features are highly relevant to the CP12 family (Singh *et al.*, 2008; Groben *et al.*, 2010; Marri *et al.*, 2010). Our results suggest that both CP12-1 and CP12-2 are essential for normal growth and development in Arabidopsis. Additionally, since it is not possible to differentiate the contribution of each individual CP12 to this phenotype and since the phenotype only becomes evident when both *CP12* transcript levels are affected, it is possible that these two genes are functionally redundant in photosynthetic tissues.

Growth analysis of the CP12 T-DNA mutant collection revealed growth reductions in some of the mutants, particularly significant for the *cp12-1/2* and *cp12-1/2/3* mutants. Our results also showed reductions in the photosynthetic performance of these plants during the early stages of development (Fig. 7). It is possible that these reductions in PSII operating efficiency are able to explain the reductions in growth observed in these plants. It has been shown before how small changes in photosynthesis are likely to have a cumulative impact on plant growth and development. A good example of this phenomena is the impact of overexpression of SBPase in tobacco plants, where an increase in carbon fixation of as little as 6–12% was found to be reflected in an up to 30% increase in leaf area and biomass (Lefebvre *et al.*, 2005; Simkin *et al.*, 2015). We propose that lack of CP12 can have a negative effect on photosynthetic performance in small seedlings which results in a slow growth phenotype sustained throughout the life of the plant.

To explore the underlying causes of the reductions in photosynthesis and given that *in vivo* in *A. thaliana* the PRK/GAPDH/CP12 complex has not been detected in either wild-type (Howard *et al.*, 2011*b*) or CP12-overexpressing transgenic plants (López-Calcagno, 2013), we looked for potential changes in the PRK and GAPDH proteins. No change in the redox state of these proteins was identified, but, unexpectedly, we detected significantly reduced levels of PRK protein in the CP12 mutant plants. This phenotype correlates with changes in the levels of the *CP12-1* and *CP12-2* transcripts. Based on results from antisense transgenic PRK plants, this reduction in PRK protein probably explains the substantial reductions in photosynthesis observed in the mature *cp12-1/2/3RNAi* plants. It has been reported that reductions in PRK activity can lead to reductions in photosynthesis; more specifically, that reductions of >85% of wild-type PRK activity levels lead to significant reductions in photosynthetic rates, but smaller reductions in PRK do not affect CO_2_ assimilation (Paul *et al.*, 1995; Habash *et al.*, 1996). Our results are consistent with these findings; *cp12-1* and *cp12-1/3* show small reductions in PRK protein levels in the order of 50–60% and reveal a mild growth phenotype. The *cp12-1/2* and *cp12-1/2/3* mutants have a more severe decrease in PRK protein (>75% less than the wild type) and, although these plants have a significant slow growth phenotype, no detectable reduction in CO_2_ assimilation was evident in the mature leaves. Finally, the *RNAi* lines, with dramatic reductions of >80% below that of wild-type levels of PRK, not only develop a severe growth phenotype but also have significant decreases in photosynthetic CO_2_ assimilation. Furthermore, line *RNAi4*, which has the lowest levels of PRK, also has the largest reduction in CO_2_ assimilation. These decreases in PRK protein, but not *PRK* transcripts, in the CP12 mutants and *cp12-1/2/3RNAi* lines suggest a post-translational level of regulation and, therefore, an additional potential role for CP12. Several pieces of evidence showing that CP12 can act as a chaperone for GAPDH preventing heat-induced aggregation and deactivation *in vitro* and protect against oxidative stress *in vivo* to both GAPDH and PRK have been published (Erales *et al.*, 2009; Marri *et al.*, 2014). It is possible that a novel function for CP12 *in vivo* is to stabilize PRK during its synthesis or protect it from degradation. No changes in PRK protein levels were detected in the *cp12-3* mutants, providing further evidence that the CP12-3 protein is unlikely to have a similar *in vivo* role to CP12-1 and CP12-2. This is consistent with the low expression levels of the *CP12-3* gene in photosynthetic tissues, as described by Marri *et al.* (2005*a*) and by Singh *et al.* (2008).

An interesting feature of the phenotype described herein is the apparent lack or reduced number of lateral roots in the *cp12-1/2/3* and *cp12-1/2/3RNAi* lines. Lack of photosynthesis and consequent reduced growth could be expected to lead to a reduced growth of the roots, but would not be expected to affect the development of lateral roots. In 2012, two reports described the presence of NTRC in non-photosynthetic plastids and its importance for lateral root formation (Ferrández *et al*, 2012; Kirchsteiger *et al*, 2012). Particularly relevant for this study, Ferrández and collaborators showed the existence of a link between chloroplast redox state and the development of lateral roots. The possibility that C12 is also involved in the crosstalk between thioredoxin and NTRC should be explored.

In conclusion, the phenotype described herein provides *in vivo* evidence of the relative importance of the individual members of the CP12 protein family in higher plants. In the first instance, it highlights the fact that the CP12 proteins have some level of functional redundancy. Our data also suggest that CP12-1 and CP12-2 play leading roles in normal development. Finally, this work also highlights the fact that the importance of CP12 is not only limited to the regulation of the CB cycle enzymes GAPDH and PRK through the formation of the GAPDH/CP12/PRK complex, but that it also is important for the maintenance of normal levels of PRK protein and hence the function of the CB cycle.

## Supplementary data

Supplementary data are available at *JXB* online.

**Table S1.** Primer details for cloning and screening and expression analysis.

**Fig. S1.** PCR analysis of genomic DNA from the CP12 mutants.

**Fig. S2.** Leaf numbers in WT and CP12 T-DNA insertion mutants.

**Fig. S3.** Seed yield of WT, CP12 insertion mutants, and RNAi plants.

**Fig. S4.** Complementation of the *cp12-1/2/3* mutant by expression of CP12-1::FLAG.

**Fig. S5** Immunoblot analysis of CP12::FLAG expression.

**Fig. S6.** CP12-2 RNAi construct.

**Fig. S7.** Lack of CP12 alters specific leaf area.

**Fig. S8.** Lateral root development is inhibited in the CP12 mutants.

**Fig. S9.** SDS–PAGE of total protein extracts of *cp12-1/2/3RNAi3*.

**Fig. S10.** SDS–PAGE of total protein extracts of *cp12-1/2/3RNAi4*.

**Fig. S11.** Lack of CP12 does not alter PRK or TK redox states.

**Fig. S12.** Photosynthetic light induction responses are unaffected in the mature or young rosettes of the CP12 mutant plants.

**Fig. S13**. *F*_q_′/*F*_m_′ is significantly decreased in *cp12-1/2/3RNAi* mature plants during a light induction treatment

## Author contributions

PEL. and AOA conducted the experiments, PEL and CAR designed the experiments and wrote the paper, and TL helped design and interpret experiments for the physiological characterization of the plants and reviewed the manuscript.

## Supplementary Material

JXB_Supplementary_Figures_S1_S13_table_S1

## Abbreviations:

A: net photosynthesis
*C*_a_: ambient CO_2_ concentration
CB cycle: Calvin–Benson cycle
*C*_i_: intracellular CO_2_ concentration
DAS: days after sowing
FBPA: fructose-1,6-bisphosphate aldolase
*F*_q_′/*F*_m_′: operating efficiency of PSII photochemistry
GAPDH: glyceraldehyde-3-phosphate dehydrogenase
*J*_max_: maximum rate of electron transport for ribulose bisphosphate regeneration
KD: knockdown
KO: knockout
Lhca1: PSI type I Chl *a*/*b*-binding protein
MDH: malate dehydrogenase
PRK: phosphoribulokinase
Rubisco: ribulose-1,5-bisphosphate carboxylase/oxygenase
SBPase: sedoheptulose-1,7-bisphosphatase
ssAGPase: small subunit of ADP-glucose pyrophosphorylase
TK: transketolase
*V*_cmax_: maximum carboxylation rates of Rubisco.
