# Supplementary material for: Arabidopsis CP12 mutants have reduced levels of phosphoribulokinase and impaired function of the Calvin–Benson cycle
Source: J Exp Bot. 2017 Apr 20;68(9):2285–98. doi: 10.1093/jxb/erx084 (PMC5447874; doi:10.1093/jxb/erx084)
Supplement: JXB_Supplementary_Figures_S1_S13_table_S1 [file erx084_suppl_JXB_Supplementary_Figures_S1_S13_table_S1.pdf]

application	gene	forward primer	reverse primer
gene specific PCR	CP12-1	CAAGCTTTATGAAAGCGCATC	ACGACACTCATCAGTCTCAGG
insertion specific PCR	CP12-1	ACGACACTCATCAGTCTCAGG	GCGTGGACCGCTTGCTGCAACT
gene specific PCR	CP12-2	TACGTCCGATATCCCTCCTTC	TCAGCTTTAGGAATCGTGAGC
insertion specific PCR	CP12-2	ATATTGACCATCATACTCATTGC	TCAGCTTTAGGAATCGTGAGC
gene specific PCR	CP12-3	TTCATCAGTCAGTATCGATGGG	AACACGATGAACAACGGTTTC
insertion specific PCR	CP12-3	AACACGATGAACAACGGTTTC	GCCTTTTCAGAAATGGATAAATAGCC TTGCTTC
gene specific PCR and RT-PCR	CP12-1	TCTCCTCCATCAAAATCCTCCGAC	CGAACCAAACACATTATGAAACA
gene specific PCR and RT-PCR	CP12-2	CGTGTACCTTCTCCATCCAA	TGGCTTTTGTATTCGTTTATGG
gene specific PCR and RT-PCR	CP12-3	GAGTGTTGCTTCCGTCACAA	TCATTCCAAATCAATCGGTGT
gene specific PCR and RT-PCR	Elongation factor	GTTTCACATCAACATTGTGGTCATTGG	GAGTACTTGGGGGTAGTGGCATCC
qPCR	CP12-1	GAAGCGGATGGTTGTGGTT	CTCTTCTCCACCTTCTCCGATA
qPCR	CP12-2	TTCACAGGCTGCCGTGTACC	GACGAAGACACGCTGGGTTG
qPCR	CP12-3	AGCCTGATGATGGTGACGAAGG	TCGCAAACCTCTGTCGCTTCC
qPCR (House keeping)	Cyclophylin	TCTTCCTCTTCGGAGCCATA	AAGCTGGGAATGATTCGATG
qPCR (House keeping)	Elongation factor	AGATCAACGAGCCCAAGA	CCGTTCCAATACCACCAAT
qPCR (House keeping)	Actin2	ACCTTGCTGGACGGACCTTACTGAT	GTTGTCTCGTGGATTCCAGCAGCTT
Gateway cloning	NP-CP12-1	CACCAATATCAGGAAAAATATCGGTAG TGG	ATTATCATAAGTACGACACTCATC

Supplementary Table S1.- Primer details for cloning and screening and expression analysis.

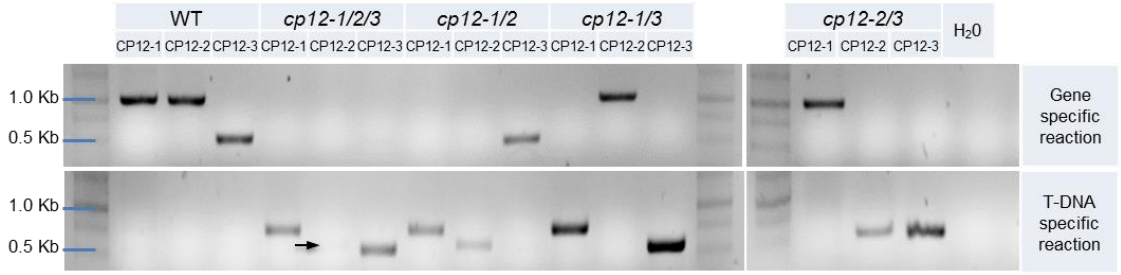

Supplementary Fig. S1.- PCR analysis of genomic DNA from the CP12 mutants. PCR analysis to confirm the presence of CP12 native alleles (upper gels, using gene specific reactions) and T-DNA insertions (bottom gels, using T-DNA specific reactions) in WT, triple *cp12-1/2/3 and* double *cp12-1/2, cp12-1/3, cp12-2/3* homozygous plants. Lanes in each genotype: **(CP12-1)** CP12-1 gene specific & T-DNA insertion specific primers were used. **(CP12-2)** CP12-2 gene specific & T-DNA insertion specific primers were used primers were used. **(CP12-3)** CP12-3 gene specific primers & T-DNA insertion specific primers were used. 1kb DNA ladder. Black arrow indicates faint band

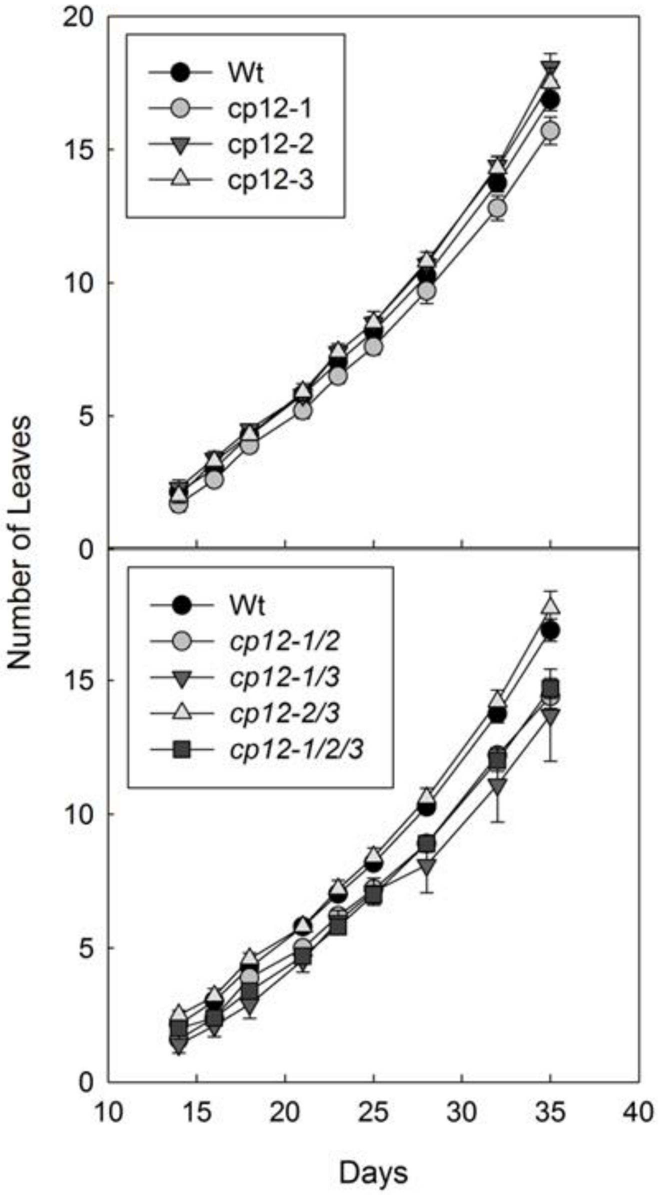

Supplementary Fig. S2. Leaf numbers in WT and CP12 T-DNA insertion mutants. Plants were grown for 7 weeks in a controlled-environment growth room (8 h light/16 h dark cycle at 22 °C) and leaf number determined. *Arabidopsis thaliana* (Col-0) WT, *cp12-1, cp12-2, cp12-3, cp12-1/2, cp12-1/3, cp12-2/3 and cp12-1/2/3* mutants are shown. Mean values ± SE are indicated, n=8-10.

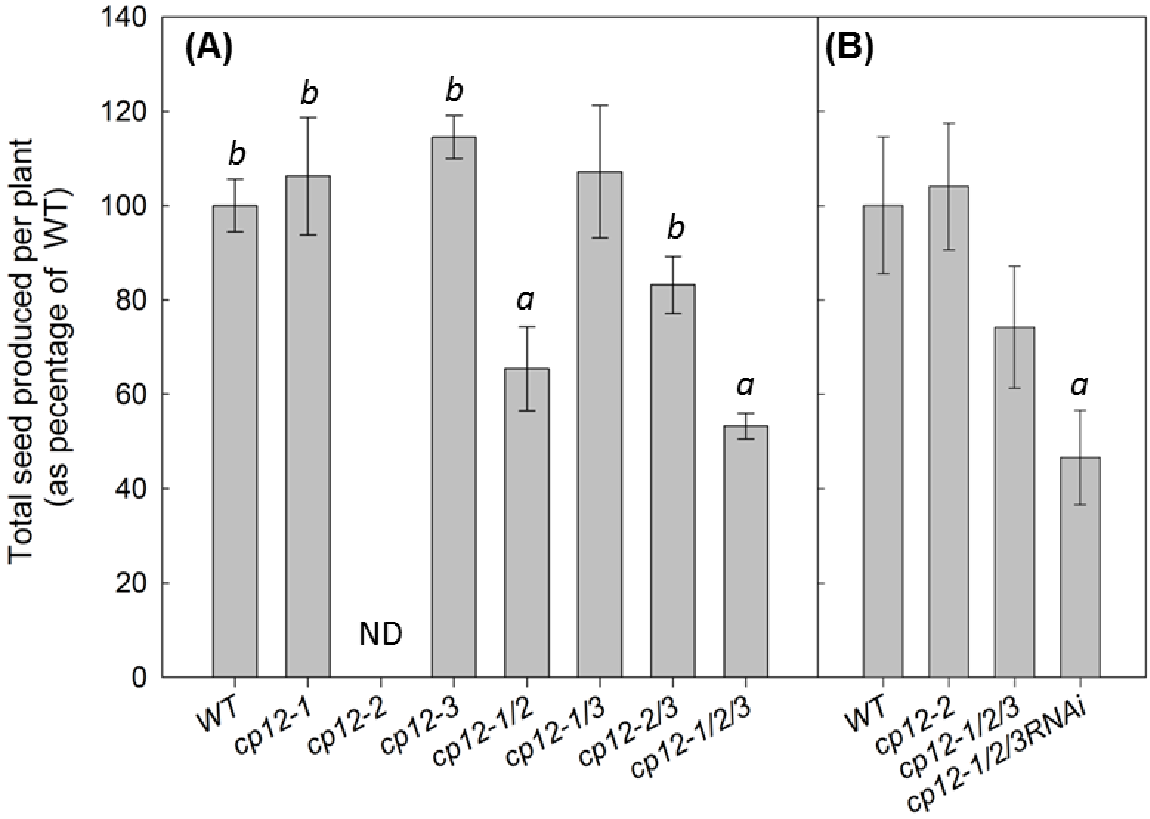

Supplementary Fig. S3. Seed yield of WT, CP12 insertion mutants and RNAi plants. (A) Total seed produced by WT and CP12 insertion mutants grown under controlled environment conditions of 8 h light/16 h dark at 22 °C. **(B)** Total seed produced by WT, *cp12-2*, *cp12-1/2/3* insertion mutants and *cp12-1/2/3RNAi* plants (*cp12-1/2/3RNAi* corresponds to lines *cp12-1/2/3RNAi3.1*, *cp12-1/2/3RNAi3.2*, *cp12-1/2/3RNAi4.1*, *cp12-1/2/3RNAi4.2* and *cp12-1/2/3RNAi4.4* no statistical differences in yield observed between these lines*)*. Mean values ± SE are indicated, n=5-30. *a*.-indicates value significantly smaller than WT, *b*.-significantly larger than *cp12-1/2/3* determined by Kruskal-Wallis 1 way ANOVA followed by Mann-Whitney test (P < 0.05). Results presented A and B correspond to two experiments.

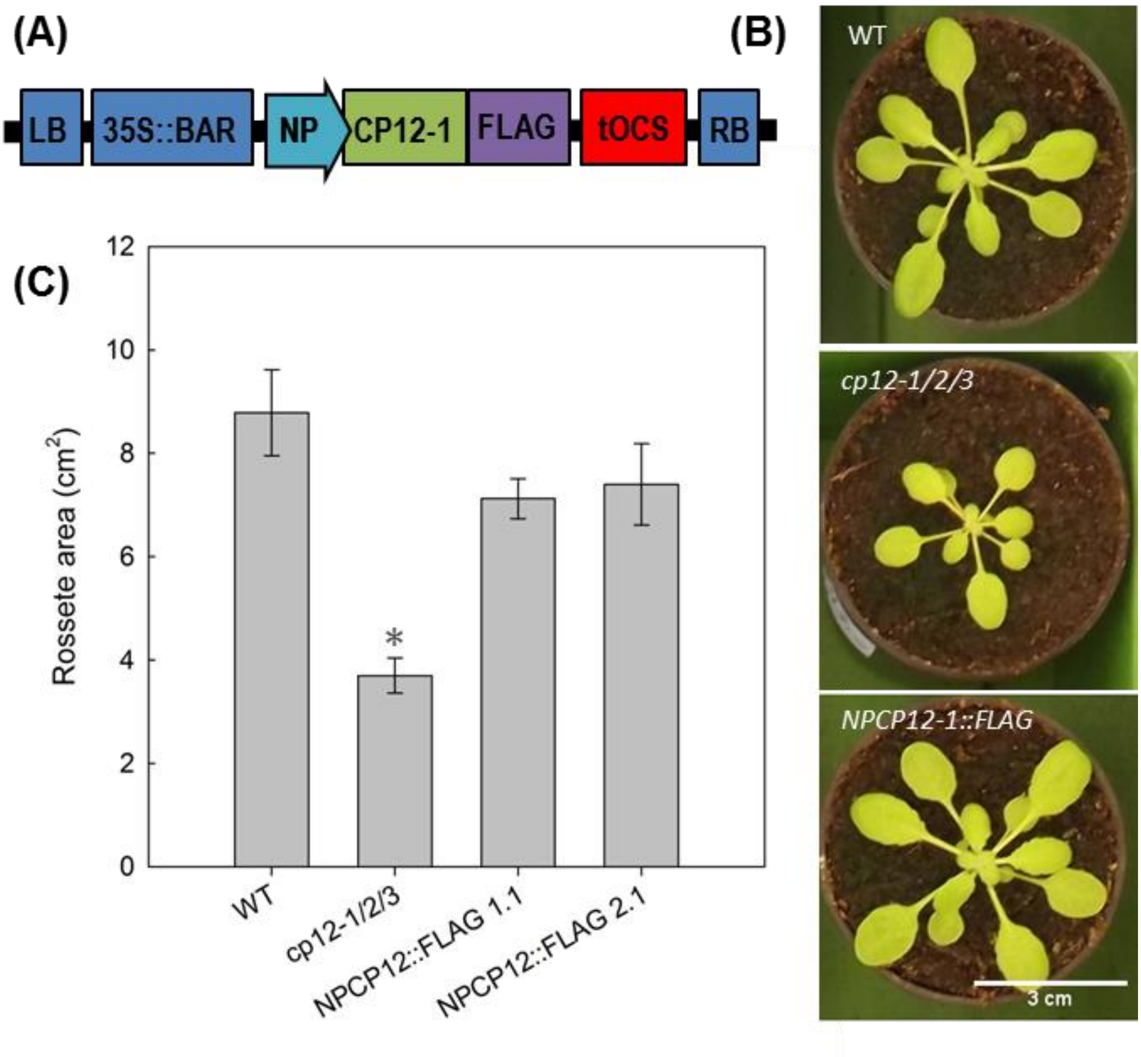

Supplementary Fig. S4. Complementation of the *cp12-1/2/3* mutant by expression of CP12-1::FLAG.

(A) Construct used for complementation of the *cp12-1/2/3* mutant. **(B)** WT and mutant Arabidopsis plants were grown on soil under controlled environment conditions (8 h light/16 h dark at 22 ºC for seven weeks and 200 µmol m^{-2} s^{-1}). Images of the plants were taken at 29 days. **(C)** Rosette area was calculated from the images in B. Mean values ± SE are indicated, n=4-11 replicates. * indicates significant differences between WT and mutants ($P < 0.05$).

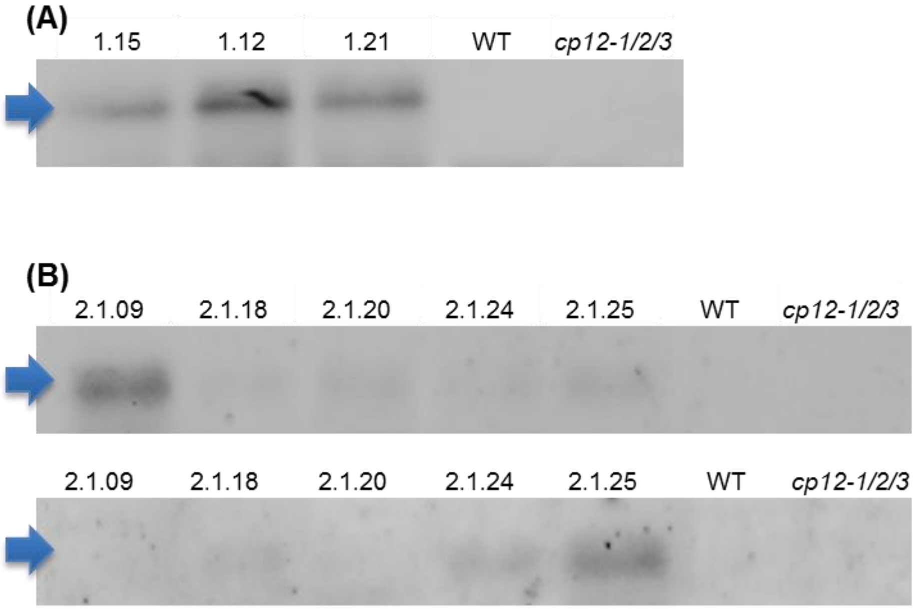

Supplementary Fig. S5 Immunoblot analysis of CP12::FLAG expression. Expression of the CP12::FLAG protein was determined in the T3 generation using western blot with antibodies against the FLAG-tag in leaf tissue protein extracts. WT and *cp12-1/2/3* extracts were used as negative controls. Two independent lines of NP-CP12-1 in the triple mutant *cp12-1/2/3* background were used **(A)** Line NPCP12-1::FLAG 1.1. **(B)** Line NPCP12-1::FLAG 2.1. The CP12-1::FLAG protein is indicated with an arrow.

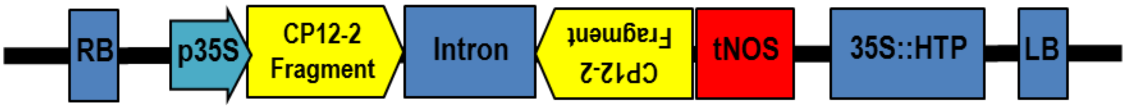

Figure S6.- CP12-2 RNAi construct

Construct used for further reducing the expression of *CP12-2* gene in *cp12-1/2/3* mutant.

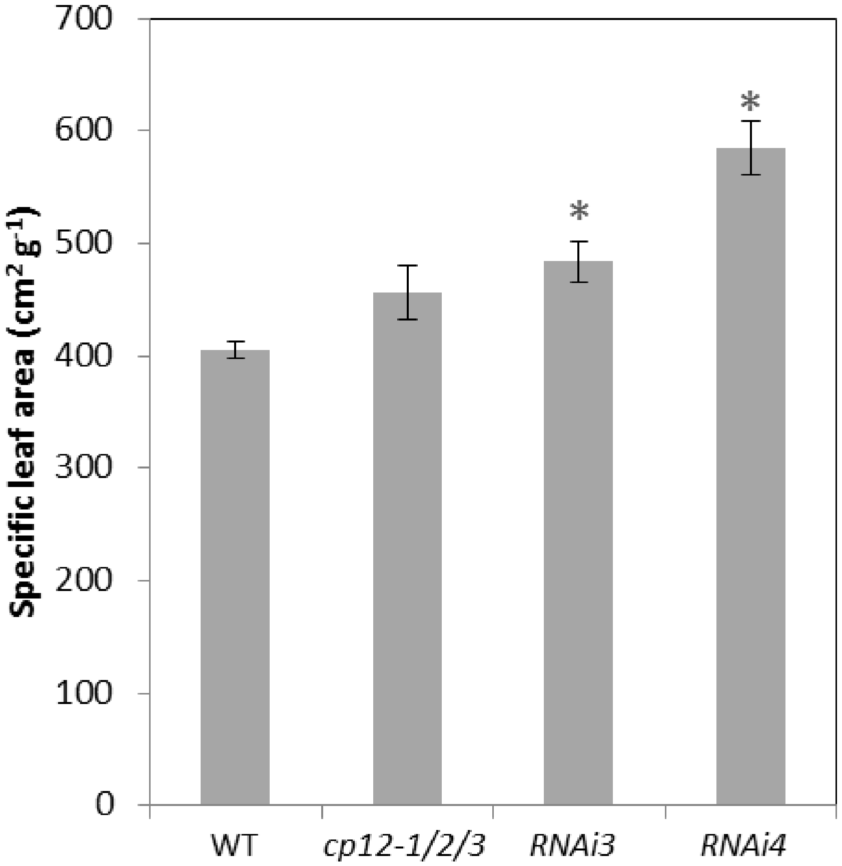

Supplementary Fig. S7.- Lack of CP12 alters specific leaf area.

Determination of specific leaf area in WT, *cp12-1/2/3* and *cp12-1/2/3RNAi* Mean values ± SE are indicated, n=5-8 replicates. * indicates significant differences between WT and mutants ($P < 0.05$).

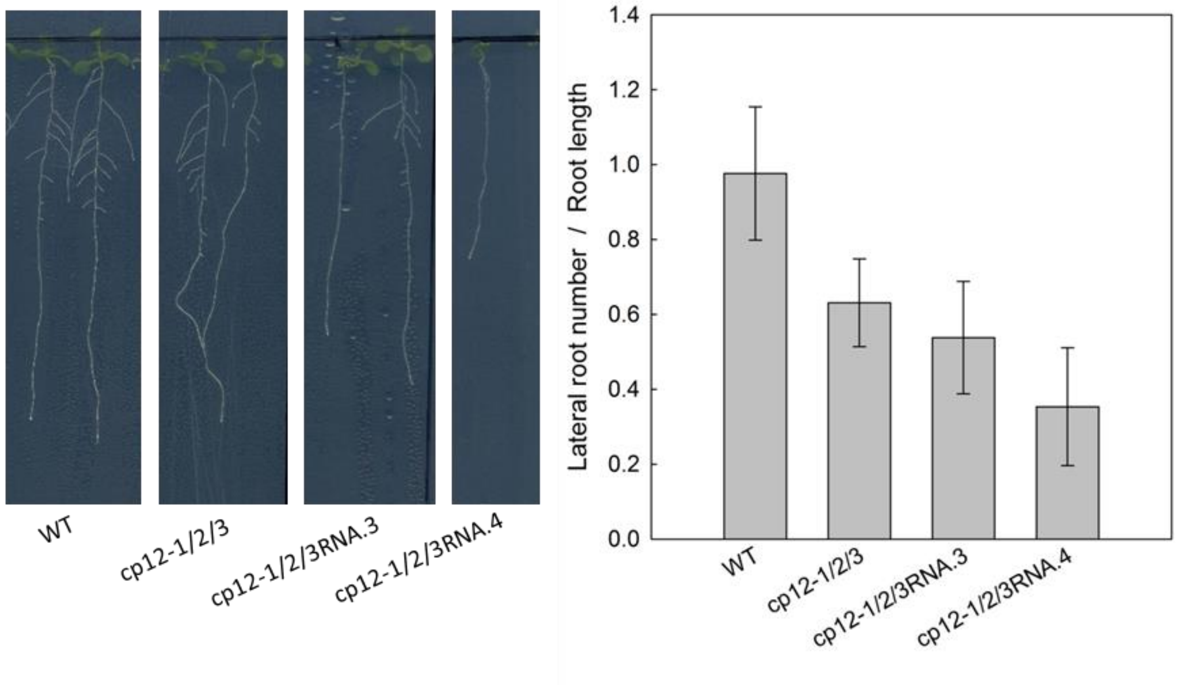

Supplementary Fig. S8.- Lateral root development is inhibited in the CP12 mutants.

(A) Impaired secondary root development evident 14 das CP12 mutant and transgenic *Arabidopsis* plants**. (B)** Quantification of the number of lateral roots related to root length of seedlings. WT, *cp12-1/2/3* and *cp12-1/2/3RNAi* lines. Plants were grown with a light intensity of 130 µmol m^{-2} s^{-1}, and 16/8 h light/dark cycle for 14 days. Mean values ± SE are indicated n=5-16 plants.

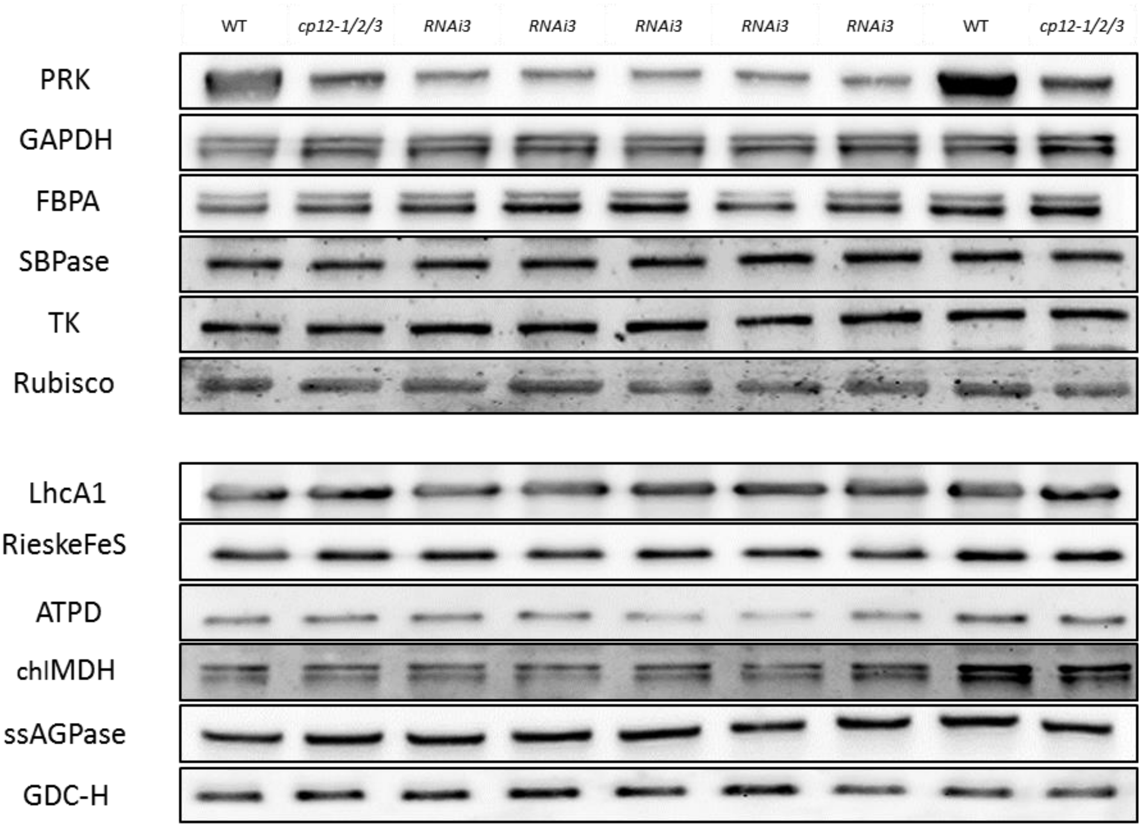

Supplementary Fig. S9.- SDS-PAGE of total protein extracts of *cp12-1/2/3RNAi3*.

Equal amounts of protein from total protein extras were loaded and separated by SDS-PAGE and then probed with a variety of antibodies against leaf proteins. A decrease in levels of PRK is evident in *cp12-1/2/3* and *cp12-1/2/3RNAi3* mutants when compared to WT. WT & *cp12-1/2/3* samples were a pool of 2-3 plants. *RNAi3* samples represent individual plants

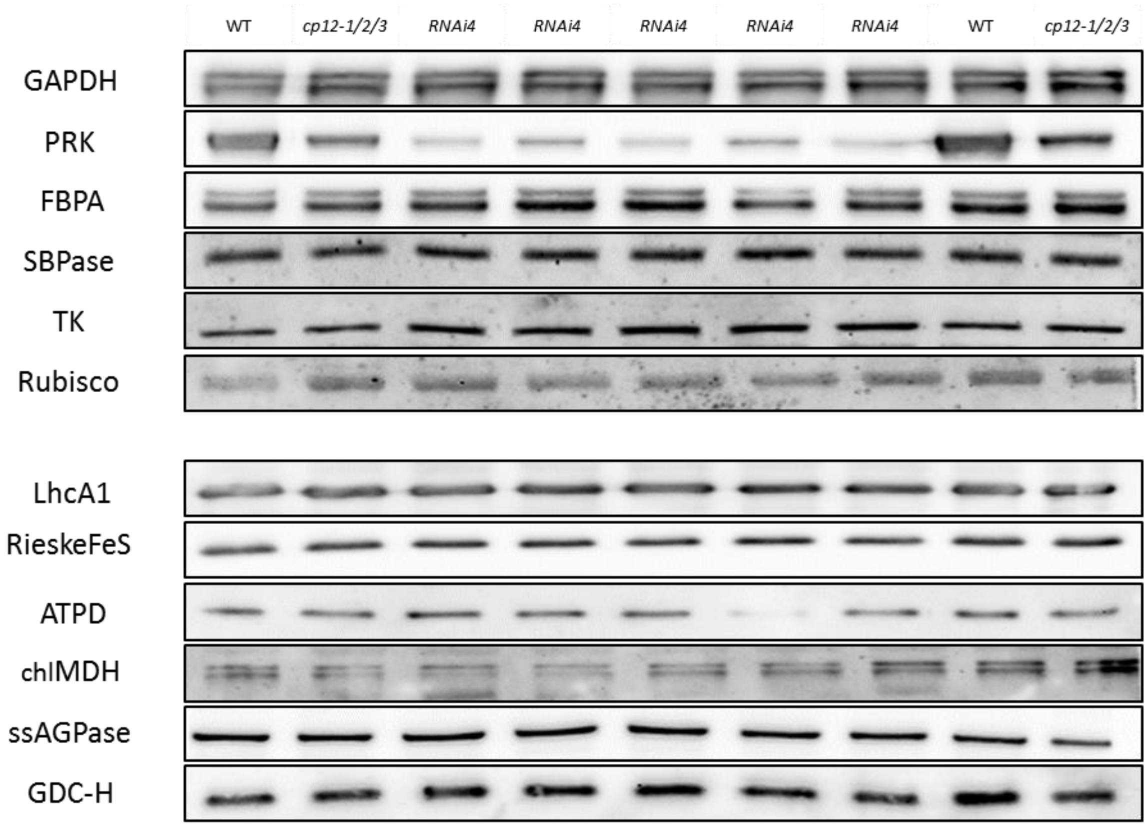

Supplementary Fig. S10.- SDS-PAGE of total protein extracts of *cp12-1/2/3RNAi4*.

Equal amounts of protein from total protein extras were loaded and separated by SDS-PAGE and then probed with a variety of antibodies against leaf proteins. A decrease in levels of PRK is evident in *cp12-1/2/3* and *cp12-1/2/3RNAi4* mutants when compared to WT. WT & *cp12-1/2/3* samples were a pool of 2-3 plants. *RNAi4* samples represent individual plants.

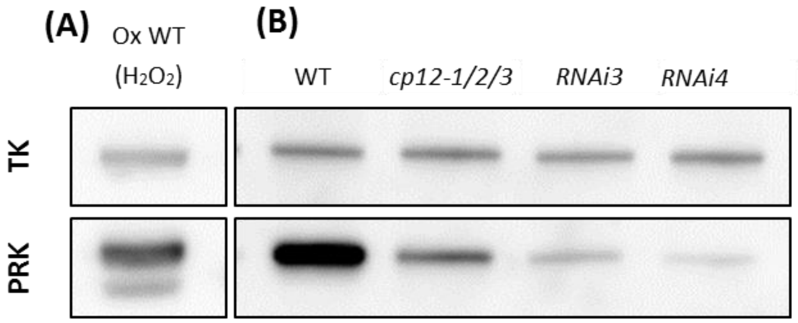

Supplementary Figure S11.- Lack of CP12 does not alter PRK or TK redox states .

Equal amounts of protein from total protein extracts were extracted and AMS treated before separating by non-reducing SDS-PAGE and then probed with antibodies against PRK and TK. Plants were grown in soil under controlled environment conditions of 8 h light/16 h dark at 22 °C, light level of 130 µmol m^{-2} s^{-1} and sampled after 4 hours of light **(A)** Before AMS treatment WT extract was treated with oxidant H_2O_2 and then proteins were separated in non-reducing SDS. **(B)** Total AMS treated-protein extracts from WT, cp12-1/2/3 and RNAi plants. Each sample represents a pool of 2-4 different plants. WT are equivalent extracts in both (A) and (B).

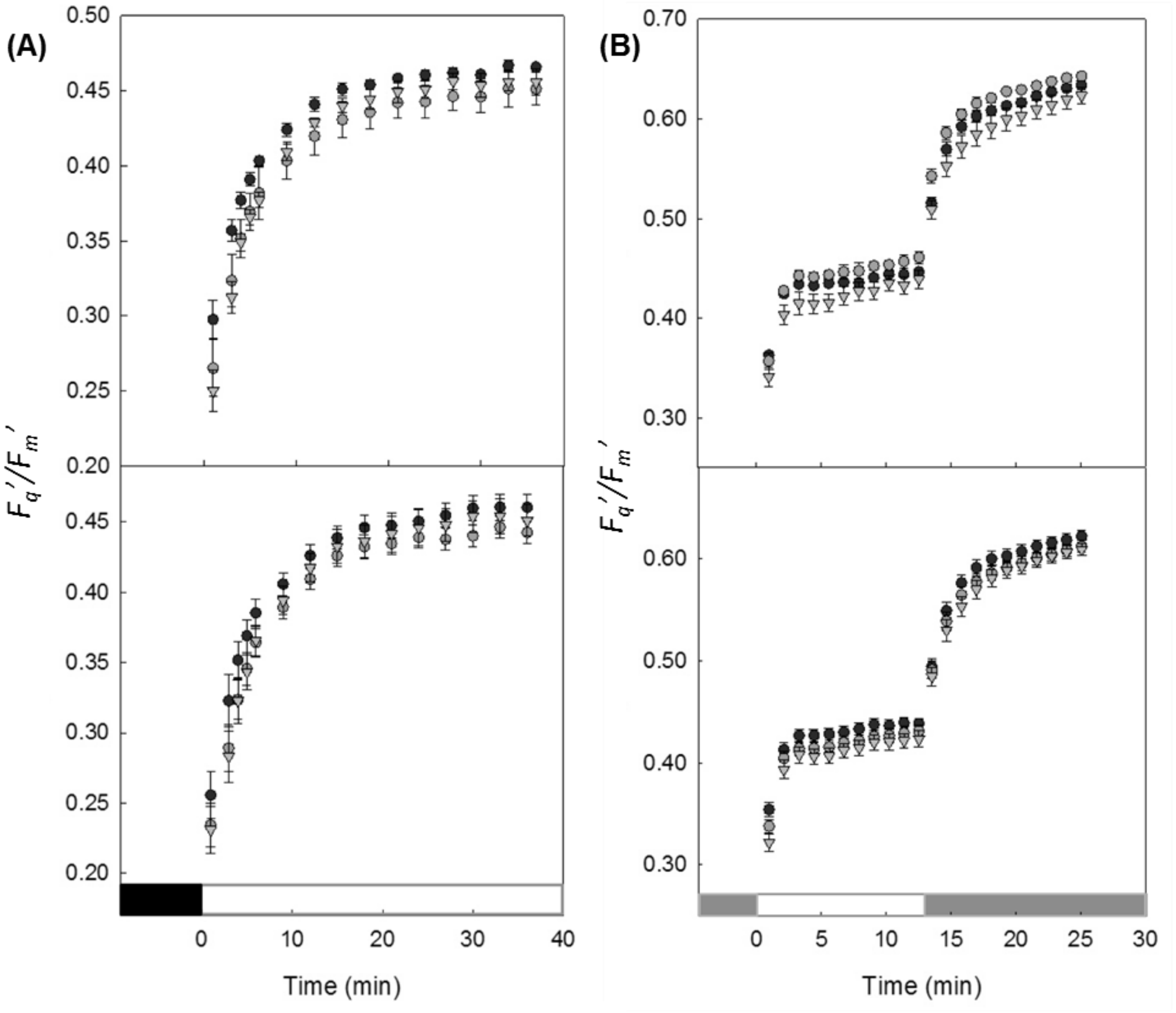

Supplementary Fig. S12. Photosynthetic light induction responses are unaffected in the mature or young rosettes of the CP12 mutant plants.

WT and CP12-mutant plants were grown in controlled environment conditions with a light intensity of 130 µmol m^{-2} s^{-1}, and 8 h light/16 h dark cycle at 22 °C and chlorophyll fluorescence was used to determine *F*q′/*F*m′ (maximum PSII operating efficiency. **(A)** F_q'/F_m' reading of plants dark adapted for 20 min followed by 400 µmol m^{-2} sec^{-1} light. **(B)** F_q'/F_m' reading of plants adapted to low light (50 µmol m^{-2} sec^{-1}) for 20 min followed by 400 µmol m^{-2} sec^{-1} for 10 min and then returned to low light. Top graphs: mature rosettes (6-7 weeks), bottom graphs: young rosettes (4-5 weeks). Data are derived from images of F' and Fm' taken from WT (black circles), *cp12-1/2* (grey circles) and *cp12-1/2/3* (light grey triangles). Mean values ± SE are indicated, for mature rosettes n=3-5, for young rossettes n=8.

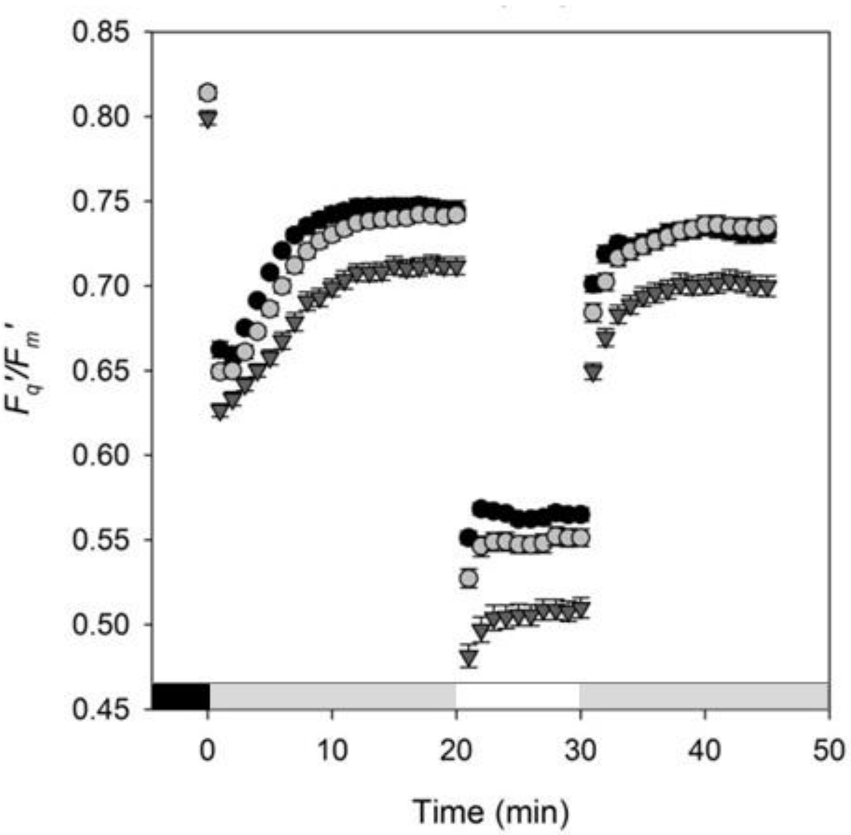

Supplementary Figure S13.- F_q'/F_m' is significantly decreased in *cp12-1/2/3RNAi* mature plants during a light induction treatment.

WT *cp12-1/2/3 and cp12-1/2/3RNAi* plants were grown in controlled environment conditions with a light intensity of 130 µmol m^{-2} s^{-1}, and 8/16 h light/dark cycle and chlorophyll fluorescence was used to determine maximum PSII operating efficiency. F_q'/F_m' *was* meassured in dark adapted plants, followed by a treatment of 20 min low light (50 µmol m^{-2} sec^{-1}) followed by 10 min high light (500 µmol m^{-2} $sec^{-1)}$ and then returned to low light. WT (black circles), *cp12-1/2/3* (grey circles) and *cp12-1/2/3RNAi* (grey triangles). Mean values ± SE are indicated, n=3-11 replicates per line. *cp12-1/2/3RNAi* represents 2 lines.
